# Supplementary material for: Ultrathin two-dimensional porous organic nanosheets with molecular rotors for chemical sensing
Source: Nat Commun. 2017 Oct 26;8:1142. doi: 10.1038/s41467-017-01293-x (PMC5656651; doi:10.1038/s41467-017-01293-x)
Supplement: Supplementary file 1 — Supplementary Information [file 41467_2017_1293_MOESM1_ESM.pdf]

Supplementary Information

Ultrathin Two-Dimensional Porous Organic Nanosheets with Molecular Rotors for Chemical Sensing

*Jinqiao Dong[1], Kang Zhang[1], Xu Li[1], Yuhong Qian[1], Hai Zhu[2], Daqiang Yuan[3], Qing-Hua Xu[2], Jianwen Jiang[1], and Dan Zhao[1]**

[1]*Department of Chemical & Biomolecular Engineering, National University of Singapore, 117585 Singapore*

[2]*Department of Chemistry, National University of Singapore, 117543 Singapore*

[3]*State Key Laboratory of Structural Chemistry, Fujian Institute of Research on the Structure of Matter, Chinese Academy of Sciences, Fuzhou, 350002 Fujian, China*

*E-mail: chezhao@nus.edu.sg

Supplementary Methods

Synthesis of 1,2-bis(4-bromophenyl)-1,2-diphenylethene (TPE-1). TPE-1 linker was synthesized following the procedure reported[1]. A mixture of 4-bromobenzophenone (2.61 g, 10 mmol) and zinc dust (1.95 g, 30 mmol) was placed in a 250 mL two-necked round-bottom flask. The flask was evacuated under vacuum and flushed with dry nitrogen three times. After tetrahydrofuran (THF) (80 mL) was added, the mixture was cooled down to 0 ℃, then 1.65 mL (15 mmol) of $TiCl_4$ was added dropwise using a syringe. After refluxed for 12 h, the mixture was quenched with 10% aqueous K_2CO_3 solution and filtered, the filtrate was extracted with dichloromethane (DCM) three times. Solvent was removed under reduced pressure and the residue was purified by column chromatography using n-hexane as eluent. A white powder of TPE-1 linker was obtained with a yield of 65% (1.59 g). ^1H NMR (400 MHz, $CDCl_3$): δ (ppm) 7.27-7.21 (m, 4H), 7.15-7.13 (m, 3H), 7.11-7.10 (m, 3H), 7.01-6.98 (m, 4H), 6.91-6.87 (m, 4H).

Synthesis of 1,1,2,2-tetrakis(4-ethynylphenyl)ethane (TPE-2). TPE-2 linker was synthesized following the procedure reported[2]. We have synthesized TPE-2 linker in a three-step process (Supplementary Fig. 1). First, a mixture of 4,4′-dibromobenzophenone (1.70 g, 5 mmol), $PdCl_2(PPh_3)_2$ (140.38 mg, 0.2 mmol), CuI (76.18 mg, 0.4 mmol) and PPh_3 (157.37 mg, 0.6 mmol) was placed in a 250 mL two-necked round-bottom flask. The flask was evacuated under vacuum and flushed with dry nitrogen three times. A mixture of THF (20 mL) and triethylamine (TEA) (20 mL) was then added and stirred for 1 h, then trimethylsilylacetylene (1.77 mL, 12.5

mmol) was added dropwise by a syringe. The mixture was stirred for 24 h at 50˚C. Afterwards, the precipitate was removed by filtration, and the filtrate was under reduced pressure to remove solvent and the residue was purified by column chromatography using n-hexane as eluent. A white powder of 4,4 ′-bis(trimethylsilyl)benzophenone (1) was obtained with the yield of 78% (1.46 g). Second, a mixture of compound 1 (1.12 g, 3 mmol) and zinc dust (0.59 g, 9 mmol) was placed in a 250 mL two-necked round-bottom flask. The flask was evacuated under vacuum and flushed with dry nitrogen three times. After THF (40 mL) was added, the mixture was cooled down to 0 ℃. Then 0.50 mL (4.5 mmol) of $TiCl_4$ was added dropwise using a syringe. After refluxed for 12 h, the mixture was quenched with 10% aqueous K_2CO_3 solution and filtered, the filtrate was extracted with DCM three times, then solvent was removed under reduced pressure and the residue was purified by column chromatography using n-hexane as eluent. A pale yellow powder of 1,1,2,2-tetrakis(4-(trimethylsilylethynyl)phenyl)ethene (2) was obtained in the yield of 71% (0.76 g). Lastly, compound 2 (0.72 g, 1 mmol), KOH (1.12 g, 20 mmol), and a mixture of THF (20 mL) and methanol (20 mL) was added in a 250 mL round-bottom flask. The mixture was stirred for 12 h at room temperature. After removing solvent under reduced pressure, 1 M HCl solution (20 mL) was added and then extracted by DCM three times. Afterwards, the solvent was removed and the residue was purified by column chromatography using n-hexane as eluent. A yellow powder of TPE-2 linker was obtained in the yield of 82% (0.35 g). ^{1}H NMR (400 MHz, $CDCl_3$): δ (ppm) 7.26-7.24 (d, 8H), 6.95-6.92 (d, 8H), 3.06 (s, 4H).

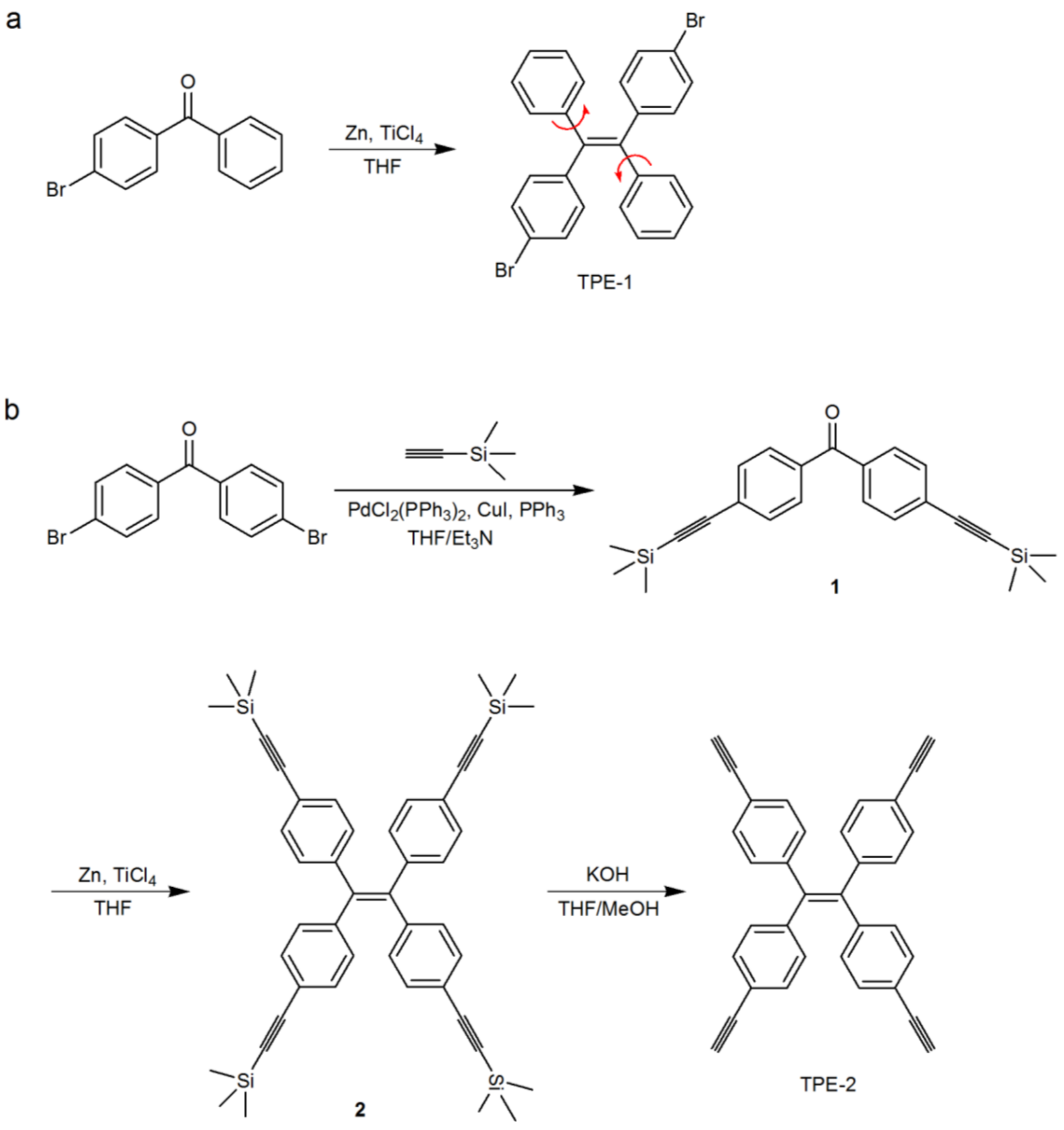

Supplementary Figure 1│Synthetic route of linkers. **a**, The synthetic route of TPE-1 linker. **b**, The synthetic route of TPE-2 linker.

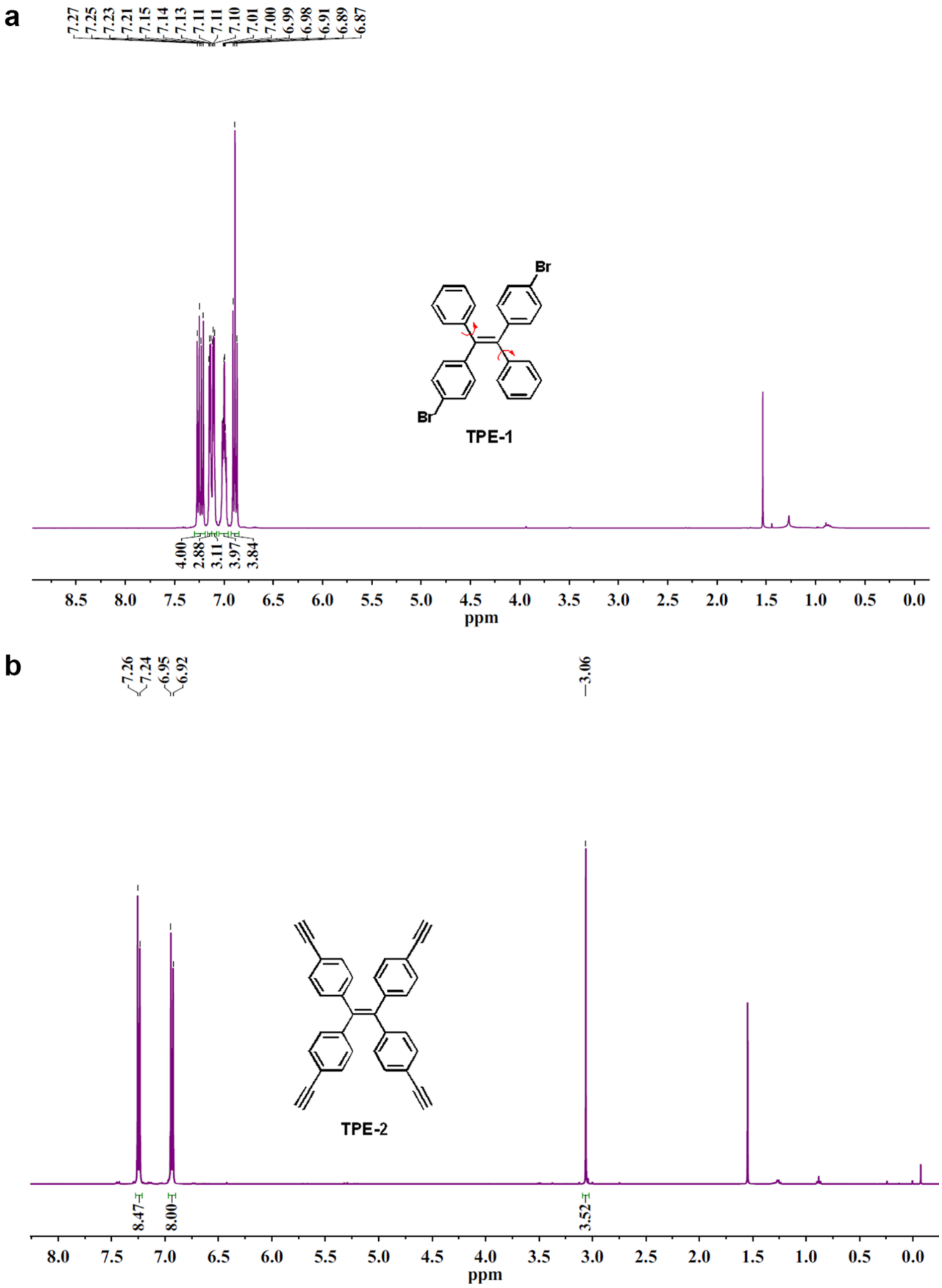

Supplementary Figure 2 | ^{1}H NMR spectra of linkers. **a**, ^{1}H NMR spectra of TPE-1 linker. **b**, ^{1}H NMR spectra of TPE-2 linker (^{1}H NMR characterization of TPE-1 and TPE-2 linkers was conducted in $CDCl_3$).

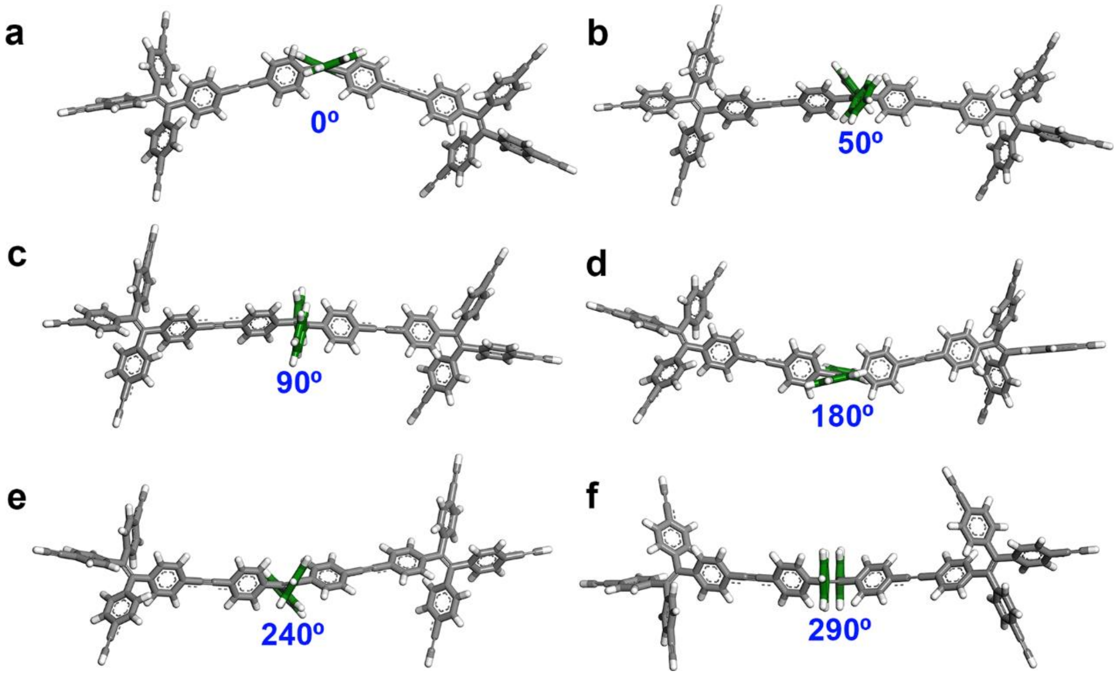

Supplementary Figure 3│The cluster models of AA stacking model of NUS-24 with different TPE rotor angles. **a**, 0 º. **b**, 50 º. **c**, 90 º. **d**, 180 º. **e**, 240 º. **f**, 290 º.

Supplementary Note 1: The cluster models were constructed for NUS-24-AA model containing one TPE-1 linker and two TPE-2 linkers. The dangling bonds in the cluster models were saturated by H atoms. The constraint optimization was carried out using the B3LYP functional with 6-31G(d) basis set. All the DFT calculations were performed by Gaussian 09[3].

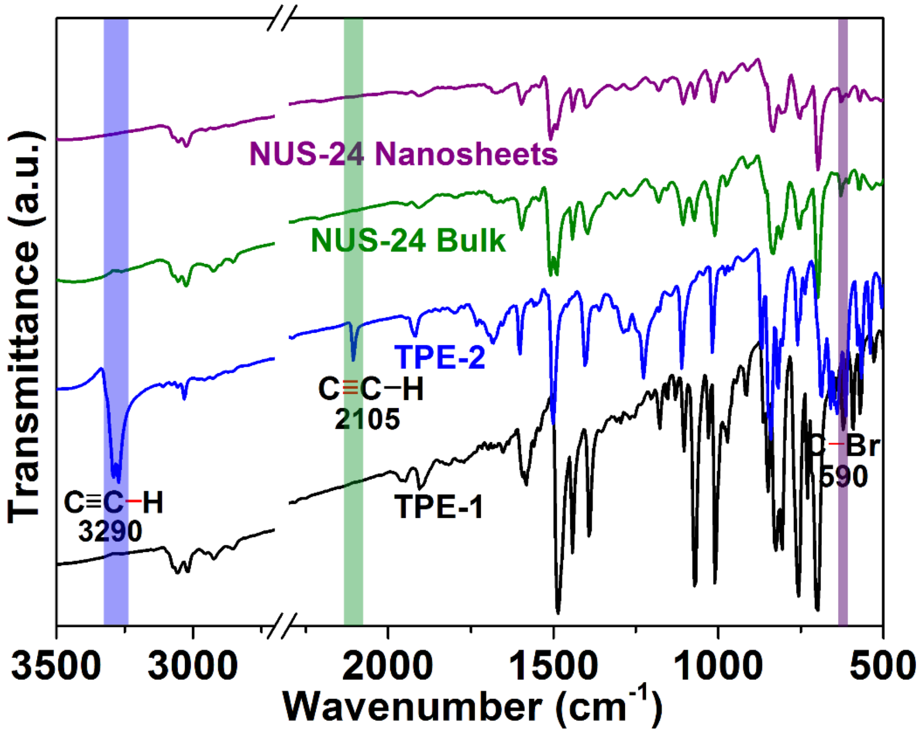

Supplementary Figure 4 | FT-IR spectra of TPE linkers, NUS-24 bulk powder and NUS-24 nanosheets.

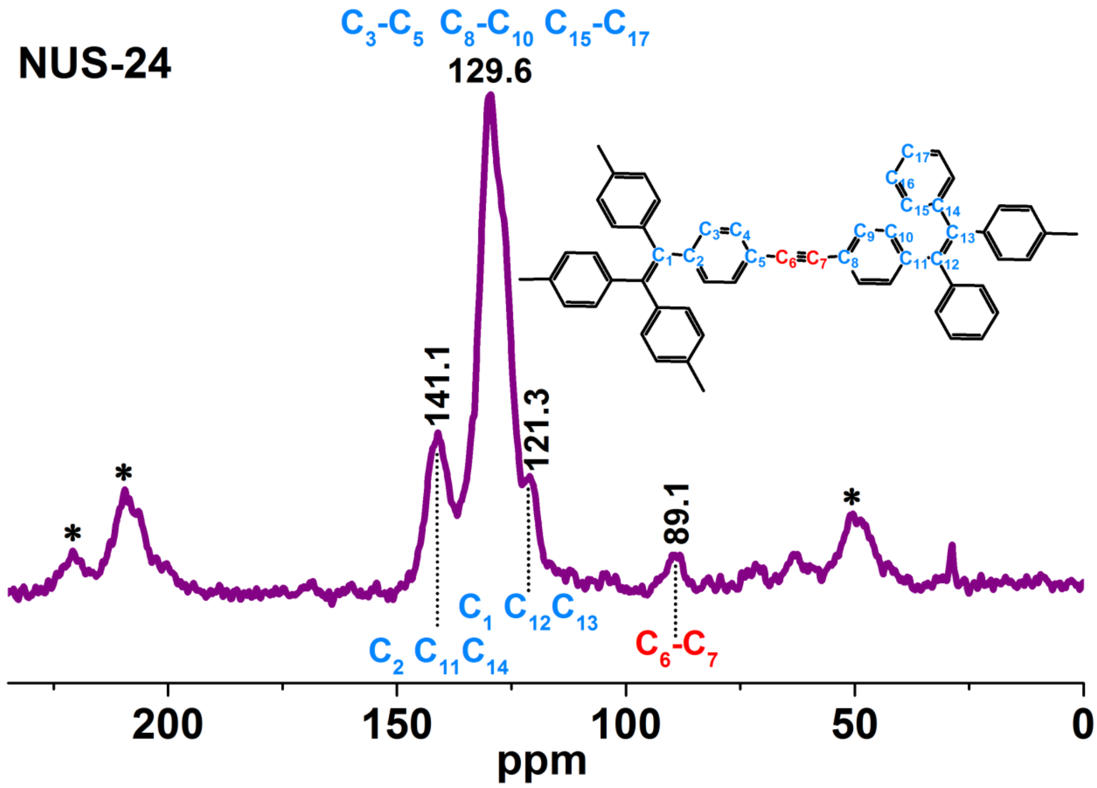

Supplementary Figure 5 | ^{13}C CP/MAS NMR spectra of NUS-24 bulk powder (Signals with * are sidebands in the test).

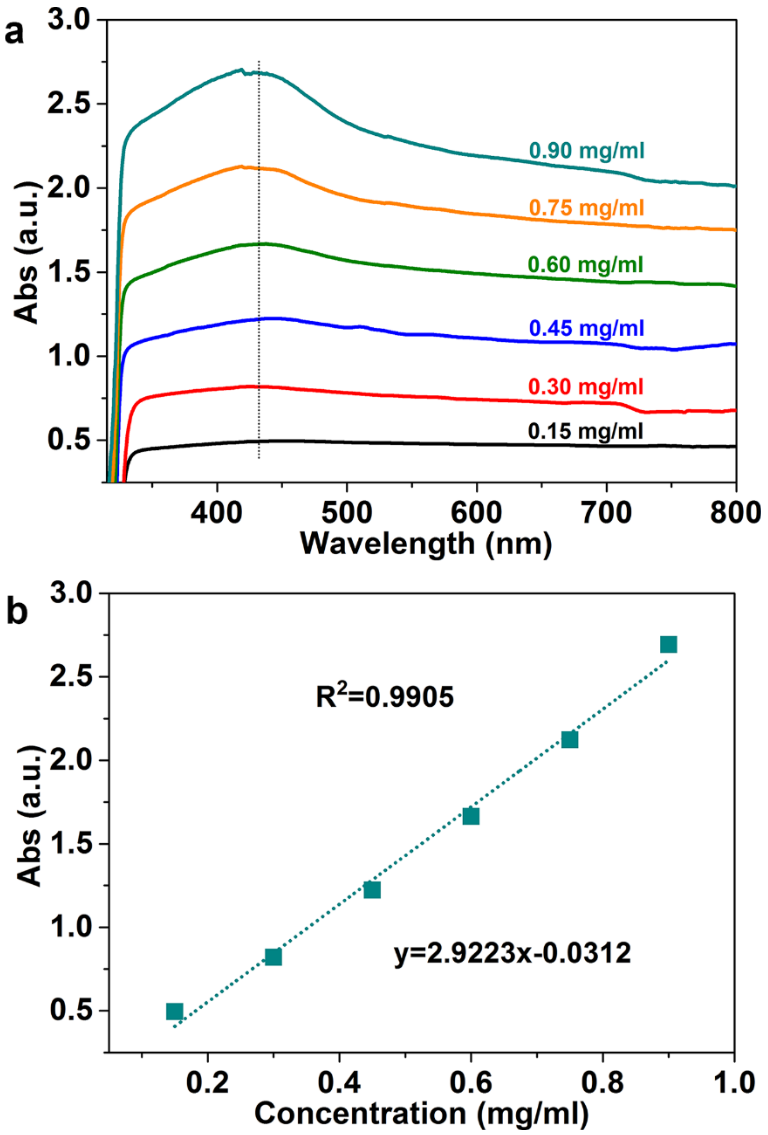

Supplementary Figure 6 | UV-Vis spectra of NUS-24 bulk powder. a, UV-Vis spectra of NUS-24 bulk powder suspended in acetone with different concentrations. **b**, The linear relationship between concentration and UV-Vis absorbance at 430 nm.

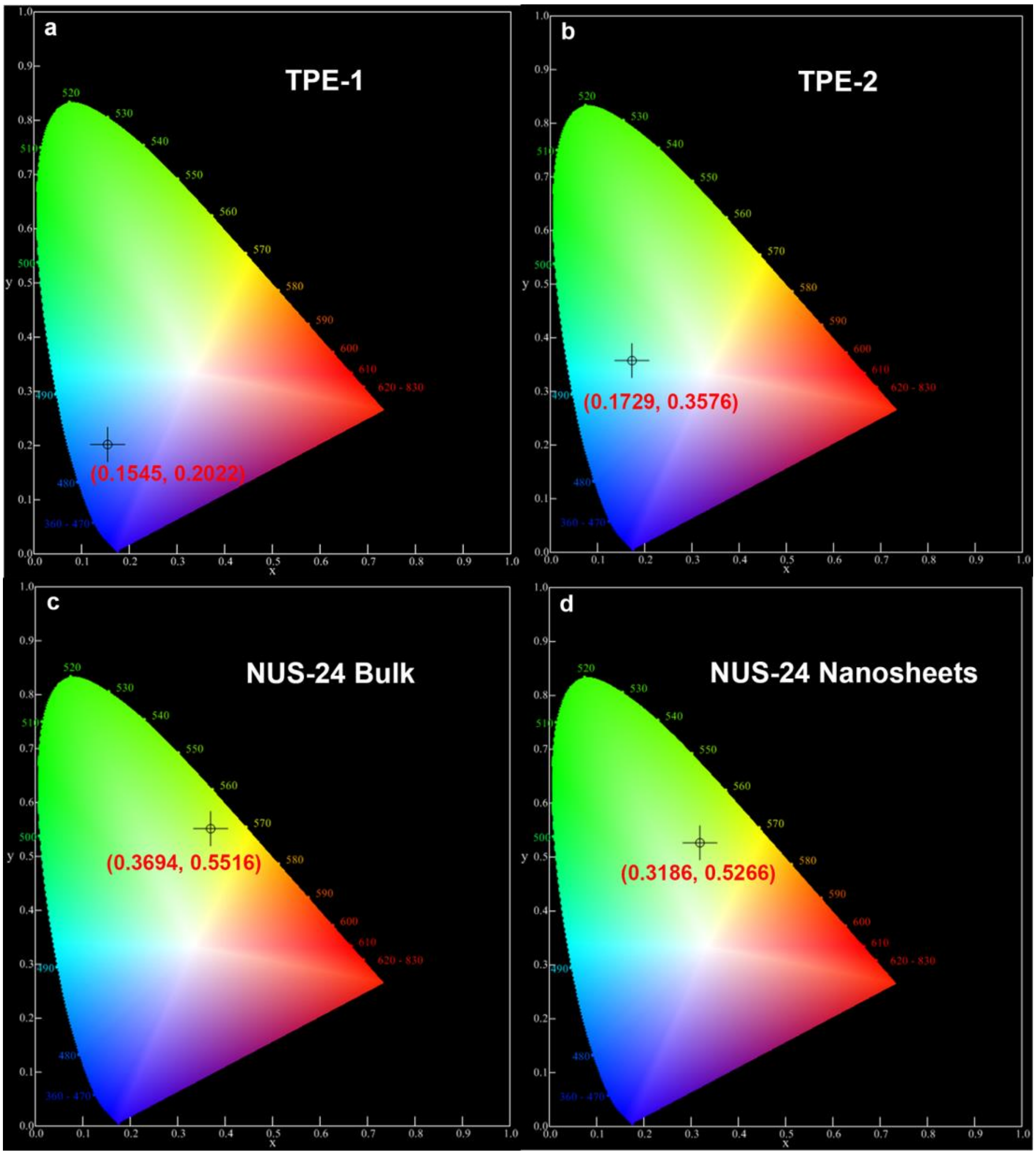

Supplementary Figure 7 | The CIE chromaticity coordinates of linkers, NUS-24 bulk powder and NUS-24 nanosheets. **a**, TPE-1 (water/acetone, 90:10, v:v). **b**, TPE-2 (water/acetone, 90:10, v:v). **c**, NUS-24 bulk powder (acetone). **d**, NUS-24 nanosheets (acetone). X is the chromaticity coordinate that represents the proportion of red primary. Y is the chromaticity coordinate that represents the proportion of green primary.

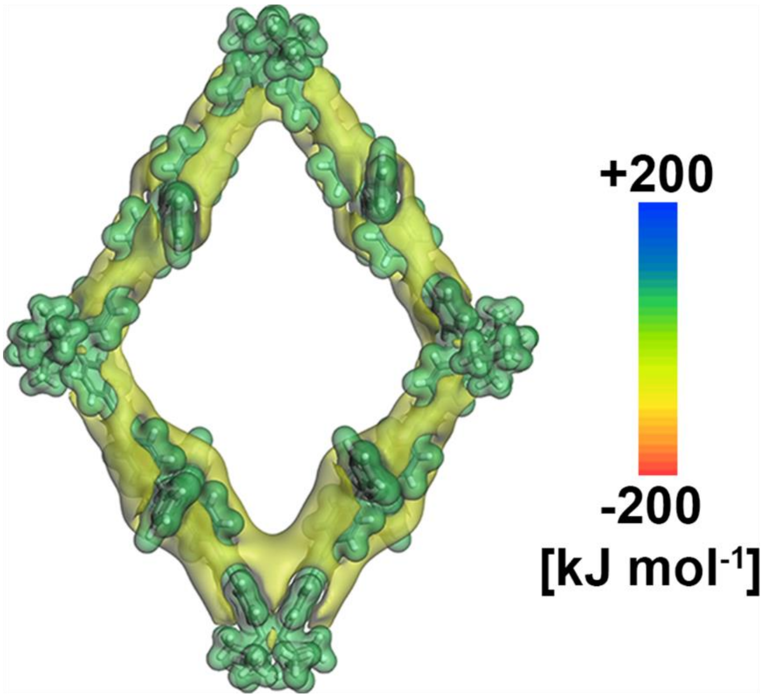

Supplementary Figure 8 | Electrostatic potential surface of NUS-24 fragment obtained by DFT calculations using DMol3 in Materials Studio.

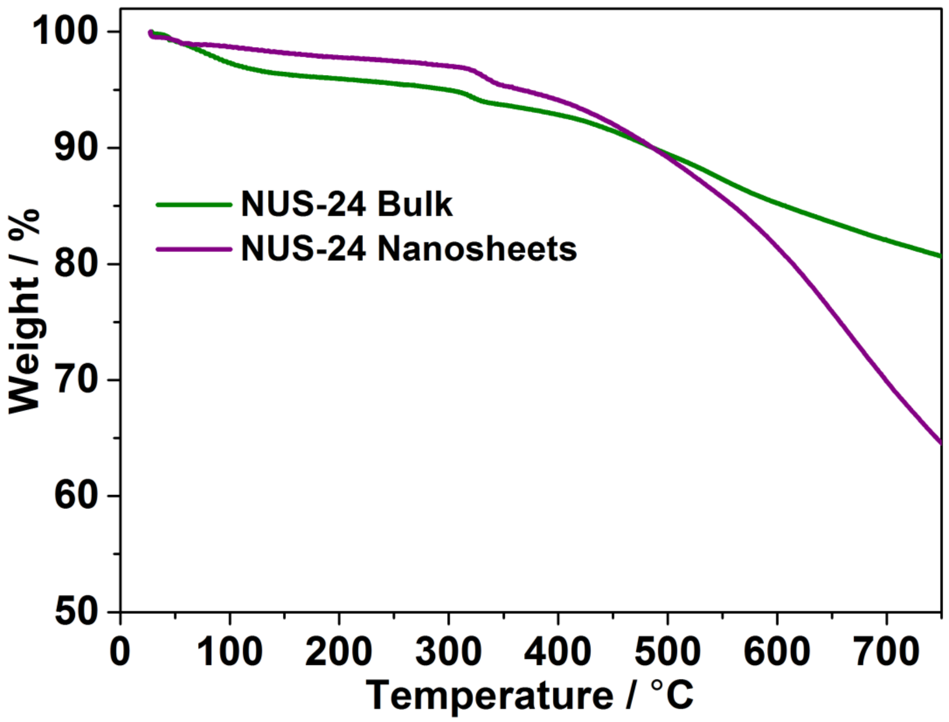

Supplementary Figure 9 | TGA curves of NUS-24 bulk powder and NUS-24 nanosheets.

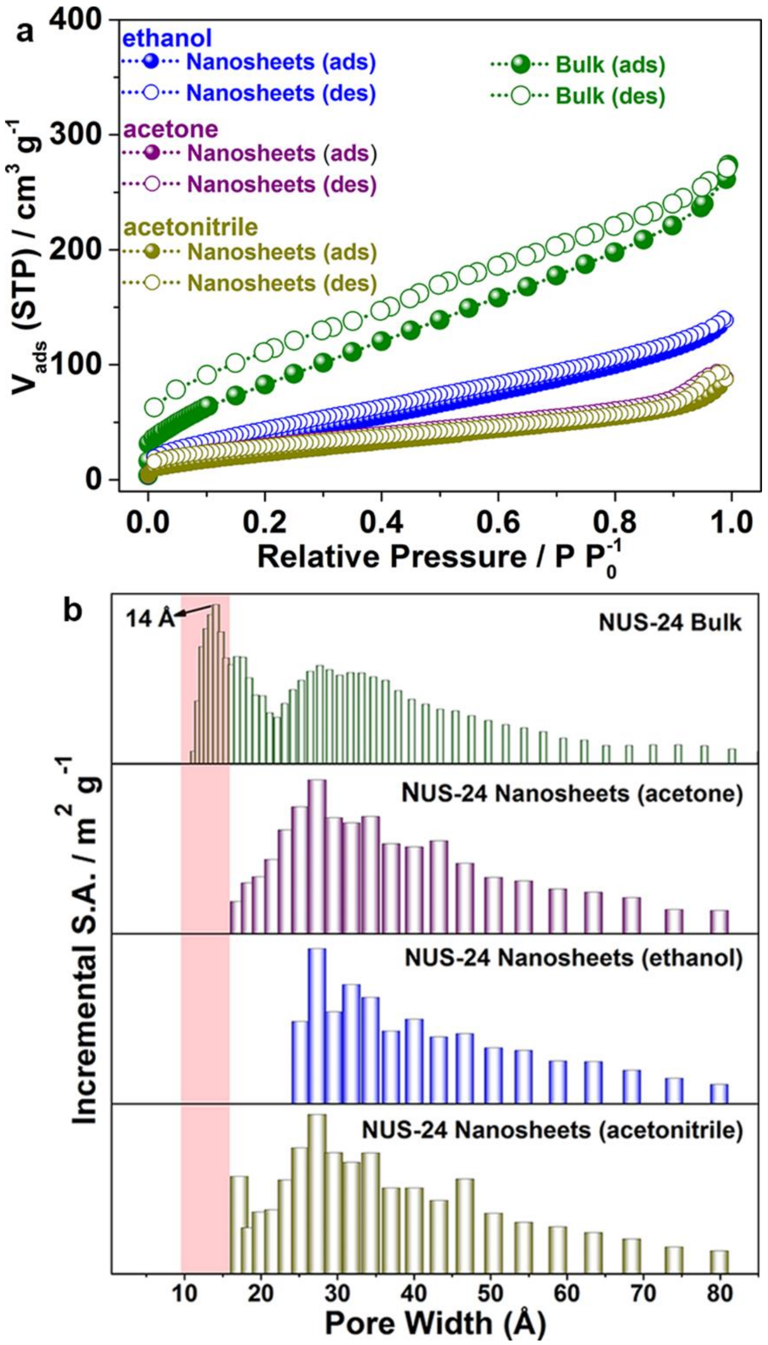

Supplementary Figure 10 | Porosity of NUS-24 bulk powder and NUS-24 nanosheets. a, N_2 sorption isotherm (filled, adsorption; open, desorption) of NUS-24 bulk powder and NUS-24 nanosheets (exfoliation by acetone, ethanol, and acetonitrile, respectively) at 77 K. **b**, The pore size distribution of NUS-24 bulk powder and NUS-24 nanosheets.

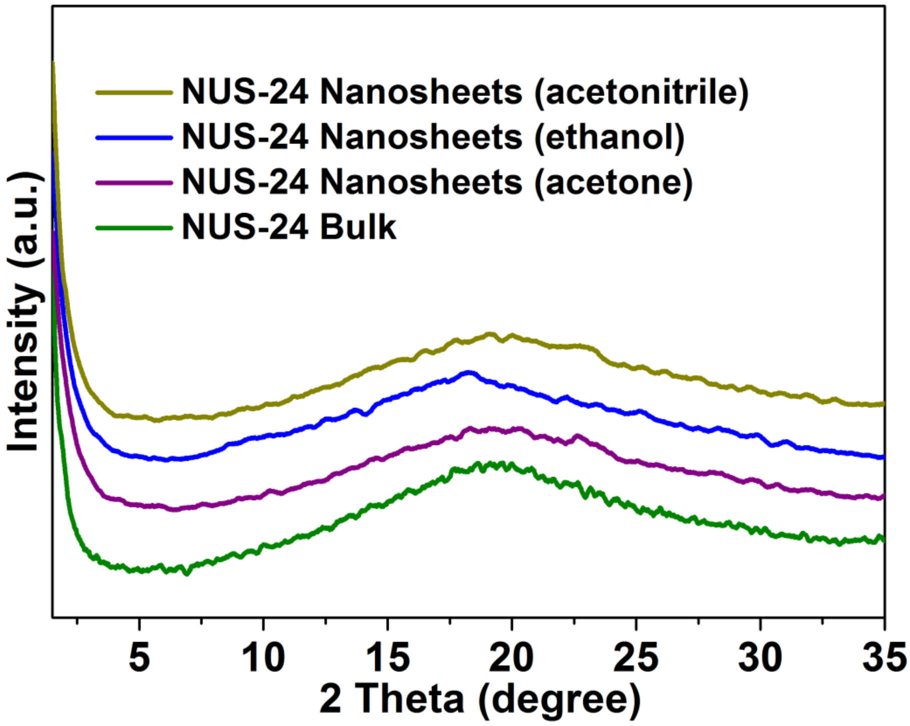

**Supplementary Figure 11 | ** PXRD patterns of NUS-24 bulk powder and NUS-24 nanosheets.

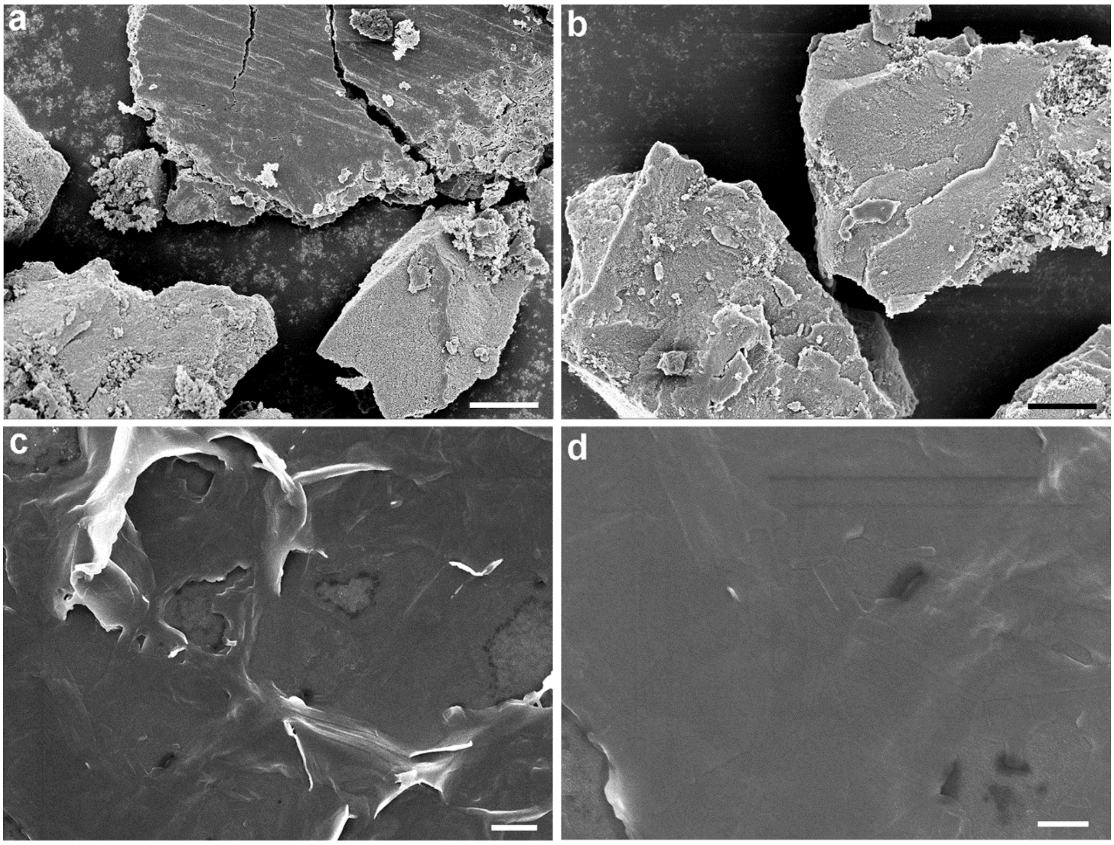

Supplementary Figure 12 | FE-SEM images of NUS-24 bulk powder and NUS-24 nanosheets. a-b, FE-SEM images of NUS-24 bulk powder (Scale bar, 10 μm in **a** and **b**). **c-d**, FE-SEM images of NUS-24 nanosheets deposited on AAO substrate (Scale bar, 2 μm in **c** and 1 μm in **d**).

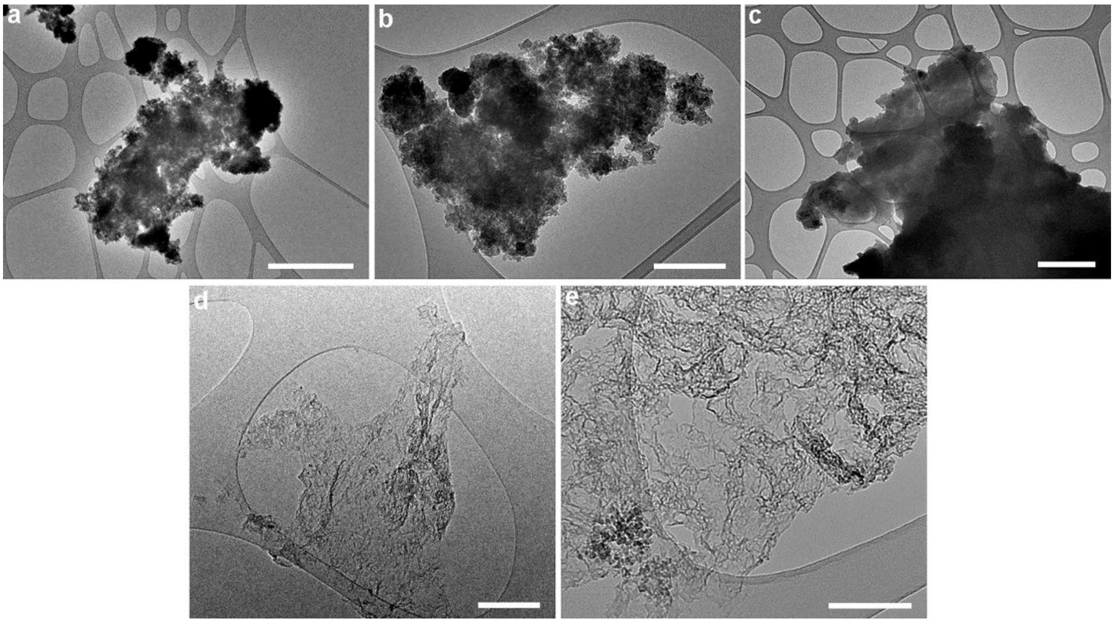

Supplementary Figure 13 | TEM images of NUS-24 bulk powder and NUS-24 nanosheets. **a**-**c**, TEM images of NUS-24 bulk powder (Scale bar, 2 μm in **a**; 500 nm in **b**; 1 μm in **c**). **d**-**e**, TEM images of NUS-24 nanosheets (Scale bar, 500 nm in **d** and 200 nm in **e**).

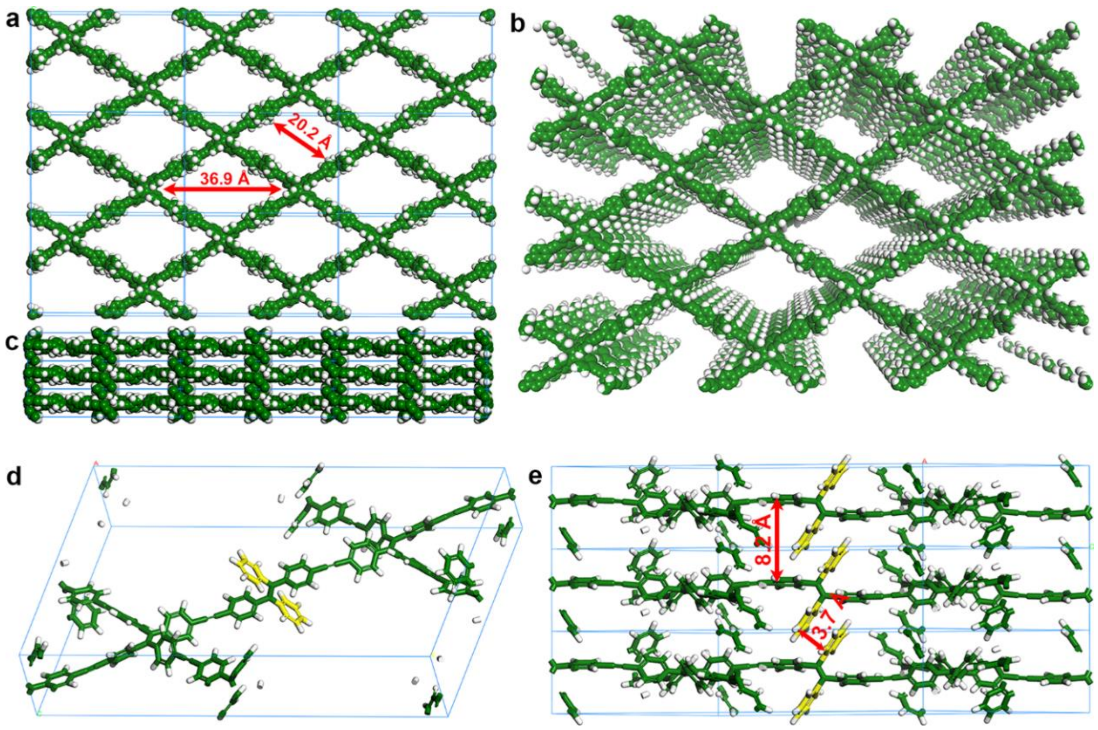

Supplementary Figure 14 | Simulated eclipsed AA stacking structure of NUS-24. **a**, The simulated crystal structure of NUS-24-AA (along *c* axis). **b**, View of the eclipsed AA stacking structure. **c**, Side view (along *b* axis) of the AA stacking structure. **d**, The dangling TPE rotors in one unit cell represented by phenyl rings with yellow colour. **e**, The distance of two adjacent layers and TPE rotors.

Supplementary Table 1 Parameters of the simulated eclipsed AA stacking structure of NUS-24 optimized by DFTB+.

**Structure parameter**	**NUS-24-AA**
Formula	$C_{141}H_{104}$
M (g mol^{-1})	1798.85
Crystal system	orthorhombic
Space group	P b a n (50)
a / Å	29.4990
b / Å	44.9898
c / Å	8.2402
α / deg.	90
β / deg.	90
γ / deg.	90
V / Å^3	10936.02
Calculated density (g cm^{-3})	0.2731
surface area (Å^2)	1781.69
Free Volume (Å^3)	8642.77
Occupied Volume (Å^3)	2293.25
Total energy (kcal mol^{-1})	540.2

Supplementary Table 2 Atomistic coordinates for the simulated AA stacking structure of NUS-24 optimized by DFTB+.

Atom	*x/a*	*y/b*	*z/c*
C1	0.29945	0.22197	0.48368
C2	0.28875	0.20123	0.35788
H3	0.26115	0.20729	0.27509
H4	0.389	0.18329	0.65312
H5	0.34447	0.23162	0.68432
C6	0.49977	-0.01379	0.27702
H7	0.48526	0.10894	0.46277
H8	0.36077	0.06302	0.35823
H9	0.5264	0.05976	0.46676
H10	0.40223	0.01379	0.35954
C11	0.31378	0.175	0.33797
H12	0.30673	0.1596	0.24026
C13	0.35065	0.168	0.44593
C14	0.36146	0.18903	0.57168
C15	0.33682	0.21552	0.58798
C16	0.42006	0.08917	0.41167
C17	0.39662	0.06197	0.38112
C18	0.41976	0.03491	0.38244
C19	0.46669	0.03398	0.41345
C20	0.48989	0.06084	0.44444
C21	0.46769	0.08826	0.44252
C22	0.49122	0.00473	0.42801
C23	0.37583	0.35973	0.57121
C24	0.396	0.38263	0.58381
C25	0.54327	0.51398	0.79439
C26	0.5521	0.53132	0.93325
C27	0.51762	0.54953	0.99947
C28	0.47401	0.54928	0.92851
C29	0.46559	0.53167	0.79073
H30	0.43019	0.50055	0.25229
H31	0.41418	0.46896	0.01128
H32	0.47537	0.43641	-0.10361
H33	0.5529	0.43686	0.01988
H34	0.56855	0.4689	0.25967
C35	0.27331	0.25	0.5

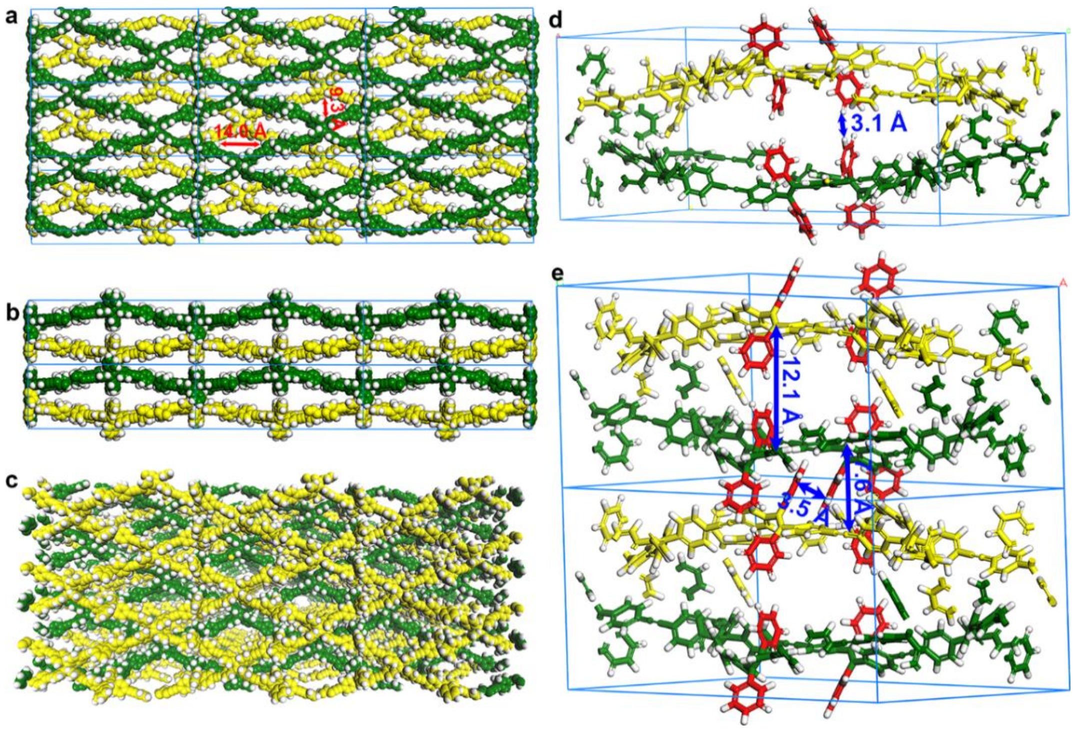

Supplementary Figure 15 | Simulated AB stacking structure of NUS-24. a, The simulated crystal structure of NUS-24-AB (along *b* axis). **b**, Side view (along *a* axis) of the AB stacking structure. **c**, View of the staggered AB stacking structure. **d**, The dangling TPE rotors in one unit cell represented by phenyl rings with red colour. **e**, The distance of two adjacent layers and TPE rotors.

Supplementary Table 3 Parameters of the simulated staggered AB stacking structure of NUS-24 optimized by DFTB+.

**Structure parameter**	**NUS-24-AB**
Formula	$C_{282}H_{208}$
M (g mol^{-1})	3597.71
Crystal system	orthorhombic
Space group	P n n a (52)
a / Å	21.8359
b / Å	18.5797
c / Å	48.5477
α / deg.	90
β / deg.	90
γ / deg.	90
V / Å^3	19696.02
Calculated density (g cm^{-3})	0.3033
surface area (Å^2)	3847.16
Free Volume (Å^3)	15027.15
Occupied Volume (Å^3)	4668.87
Total energy (kcal mol^{-1})	1831.5

Supplementary Table 4 Atomistic coordinates for the simulated AB stacking structure of NUS-24 optimized by DFTB+.

**Atom**	***x/a***	***y/b***	***z/c***
C1	0.6585	0.23013	0.27565
C2	0.64924	0.16176	0.28822
H3	0.61807	0.12285	0.27861
H4	0.76265	0.29901	0.32225
H5	0.71059	0.33054	0.27825
C6	0.85726	0.05784	-0.49927
H7	0.86121	0.22172	0.40327
H8	0.70282	0.08144	0.41431
H9	0.8878	0.20619	0.45281
H10	0.73039	0.06513	0.46369
C11	0.67963	0.14303	0.31279
H12	0.67179	0.09002	0.32207
C13	0.71953	0.19314	0.32587
C14	0.73122	0.26029	0.31271
C15	0.70162	0.27804	0.28779
C16	0.77977	0.15308	0.4052
C17	0.74354	0.10835	0.42259
C18	0.75922	0.09917	0.45055
C19	0.81084	0.13502	0.46189
C20	0.84739	0.17879	0.4444
C21	0.83238	0.18764	0.41637
C22	0.82772	0.12785	0.49213
C23	0.7454	0.32303	0.14706
C24	0.76186	0.33507	0.12346
C25	0.90193	0.47614	0.01621
C26	0.92767	0.543	0.00864
C27	0.9082	0.57832	-0.01556
C28	0.86314	0.54579	-0.03227
C29	0.83818	0.47824	-0.02511
H30	0.69361	0.3124	0.00572
H31	0.63148	0.20545	0.01954
H32	0.67949	0.0829	0.02171
H33	0.79014	0.06759	0.00917
H34	0.85255	0.17352	-0.00554
C35	0.52381	0.25834	0.22342
C36	0.53312	0.20818	0.20167
H37	0.56472	0.16296	0.20469
H38	0.41831	0.3679	0.1909

H39	0.47174	0.35361	0.23583
C40	1.27809	0.25128	0.00072
H41	0.29085	0.28492	0.10573
H42	0.4765	0.31585	0.07478
H43	1.25045	0.29458	0.0579
H44	0.43542	0.32892	0.0272
C45	0.50195	0.2152	0.17639
H46	0.50971	0.17549	0.16007
C47	0.46108	0.27375	0.17188
C48	0.45014	0.32286	0.19385
C49	0.48056	0.31464	0.21932
C50	0.38672	0.29963	0.09383
C51	0.42698	0.31219	0.07127
C52	0.40363	0.31964	0.04434
C53	0.33977	0.31332	0.03903
C54	0.29984	0.29983	0.06157
C55	0.32271	0.29425	0.08865
C56	0.31557	0.31606	0.00987
C57	0.43223	0.21657	0.35511
C58	0.41127	0.20869	0.37825
C59	1.21533	0.24082	0.5065
C60	1.18007	0.30124	-0.48571
C61	1.20685	0.3703	-0.4846
C62	0.2691	0.37875	-0.49172
C63	0.30429	0.31874	-0.50001
H64	0.41729	0.05015	-0.46478
H65	0.46284	-0.06809	0.52164
H66	0.42782	-0.13067	0.47873
H67	1.34728	-0.07355	0.44909
H68	1.30283	0.04596	0.46179
C69	0.62286	0.25	0.25
C70	0.55947	0.25	0.25

Supplementary Note 2: The structures of NUS-24-AA and NUS-24-AB were optimized first by Materials Studio with the universal force field (UFF)[4] and the QEq method[5], then further with the density-functional tight-binding (DFTB+) method incorporating dispersion interactions[6]. The DFTB+ is an approximate DFT method based on the second-order expansion of the Kohn–Sham total energy with respect to charge density fluctuations. The Slater–Koster library was set as CH, which includes the parameters for the elements of C and H. The convergence criterion of the

self-consistent charge (SCC) parameters for electronic minimization was 10^{-4} Ha. Smearing technique was used to achieve the self-consistent field convergence with a smearing value of 0.01 Ha. The lattice dimensions and shapes were optimized simultaneously.

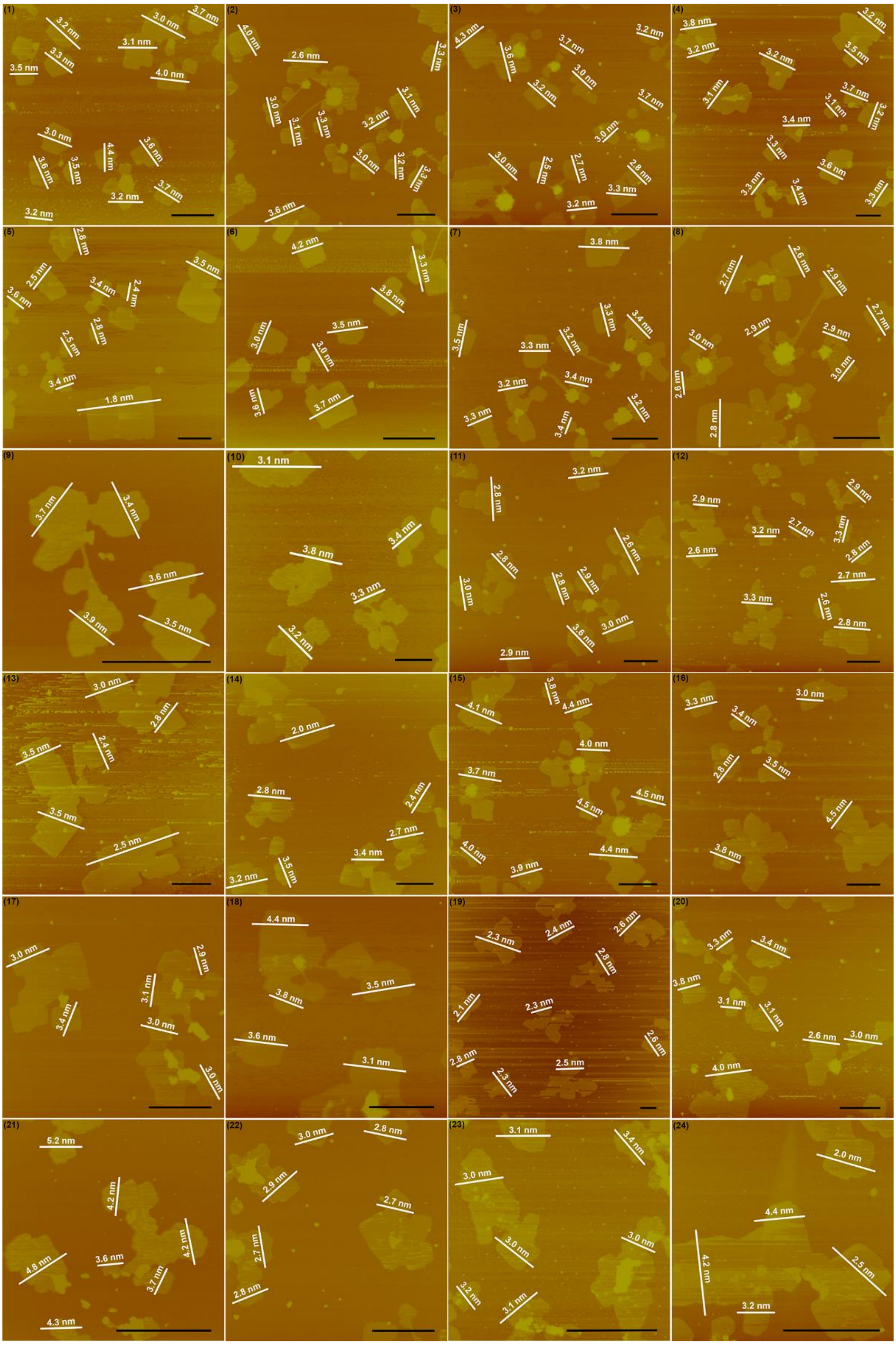

(1)
3.2 nm
3.0 nm
3.7 nm
3.1 nm
3.5 nm
3.3 nm
4.0 nm
3.0 nm
4.4 nm
3.6 nm
3.6 nm
3.5 nm
3.7 nm
3.2 nm
3.2 nm
(2)
4.0 nm
2.6 nm
3.3 nm
3.0 nm
3.1 nm
3.3 nm
3.1 nm
3.2 nm
3.0 nm
3.2 nm
3.3 nm
3.6 nm
(3)
4.3 nm
3.7 nm
3.2 nm
3.6 nm
3.0 nm
3.2 nm
3.7 nm
3.0 nm
3.0 nm
2.5 nm
2.7 nm
2.8 nm
3.3 nm
3.2 nm
(4)
3.8 nm
3.2 nm
3.2 nm
3.2 nm
3.5 nm
3.1 nm
3.7 nm
3.1 nm
3.2 nm
3.4 nm
3.3 nm
3.6 nm
3.3 nm
3.4 nm
3.3 nm
(5)
2.8 nm
3.5 nm
2.5 nm
3.4 nm
2.4 nm
3.6 nm
2.8 nm
2.5 nm
3.4 nm
1.8 nm
(6)
4.2 nm
3.3 nm
3.8 nm
3.5 nm
3.0 nm
3.0 nm
3.6 nm
3.7 nm
(7)
3.8 nm
3.3 nm
3.4 nm
3.5 nm
3.3 nm
3.2 nm
3.4 nm
3.2 nm
3.3 nm
3.4 nm
3.2 nm
(8)
2.7 nm
2.6 nm
2.9 nm
2.7 nm
3.0 nm
2.9 nm
2.9 nm
3.0 nm
2.6 nm
2.8 nm
(9)
3.7 nm
3.4 nm
3.6 nm
3.9 nm
3.5 nm
(10)
3.1 nm
3.4 nm
3.8 nm
3.3 nm
3.2 nm
(11)
3.2 nm
2.8 nm
2.6 nm
2.8 nm
3.0 nm
2.8 nm
2.9 nm
3.0 nm
3.6 nm
2.9 nm
(12)
2.9 nm
2.9 nm
3.2 nm
2.7 nm
3.3 nm
2.6 nm
2.8 nm
2.7 nm
3.3 nm
2.6 nm
2.8 nm
(13)
3.0 nm
2.8 nm
3.5 nm
2.4 nm
3.5 nm
2.5 nm
(14)
2.0 nm
2.8 nm
2.4 nm
2.7 nm
3.4 nm
3.2 nm
3.5 nm
(15)
3.8 nm
4.1 nm
4.4 nm
4.0 nm
3.7 nm
4.5 nm
4.5 nm
4.0 nm
4.4 nm
3.9 nm
(16)
3.3 nm
3.0 nm
3.4 nm
2.8 nm
3.5 nm
4.5 nm
3.8 nm
(17)
3.0 nm
2.9 nm
3.1 nm
3.4 nm
3.0 nm
3.0 nm
(18)
4.4 nm
3.5 nm
3.8 nm
3.6 nm
3.1 nm
(19)
2.3 nm
2.4 nm
2.6 nm
2.8 nm
2.1 nm
2.3 nm
2.6 nm
2.8 nm
2.5 nm
2.3 nm
(20)
3.3 nm
3.4 nm
3.8 nm
3.1 nm
3.1 nm
2.6 nm
3.0 nm
4.0 nm
(21)
5.2 nm
4.2 nm
4.2 nm
4.8 nm
3.6 nm
3.7 nm
4.3 nm
(22)
3.0 nm
2.8 nm
2.9 nm
2.7 nm
2.7 nm
2.8 nm
(23)
3.1 nm
3.4 nm
3.0 nm
3.0 nm
3.0 nm
3.2 nm
3.1 nm
(24)
2.0 nm
4.4 nm
4.2 nm
2.5 nm
3.2 nm

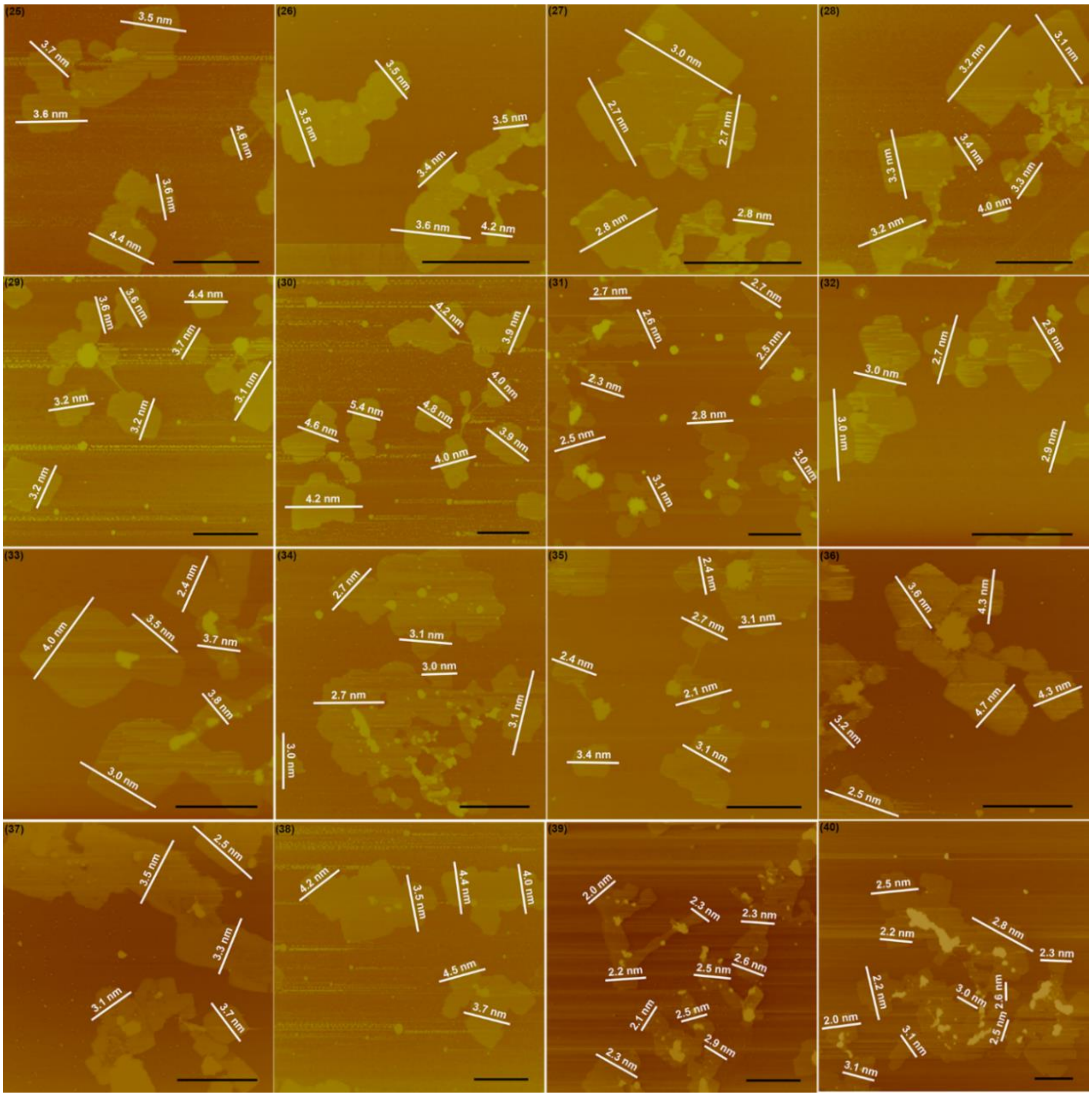

Supplementary Figure 16 | AFM images of NUS-24 nanosheets. Statistical analysis of the AFM images (322 AFM measurements from 40 pictures) of exfoliated NUS-24 nanosheets for thickness is presented in Fig. 3m in the main text. All scale bars are 2 μm.

Supplementary Note 3: 90 % of the exfoliated NUS-24 nanosheets have sheet-like morphology with a thickness of 2 - 5 nm. The lateral size of the exfoliated NUS-24 nanosheets can be as large as 6 μm shown in Supplementary Figure **16** (#37).

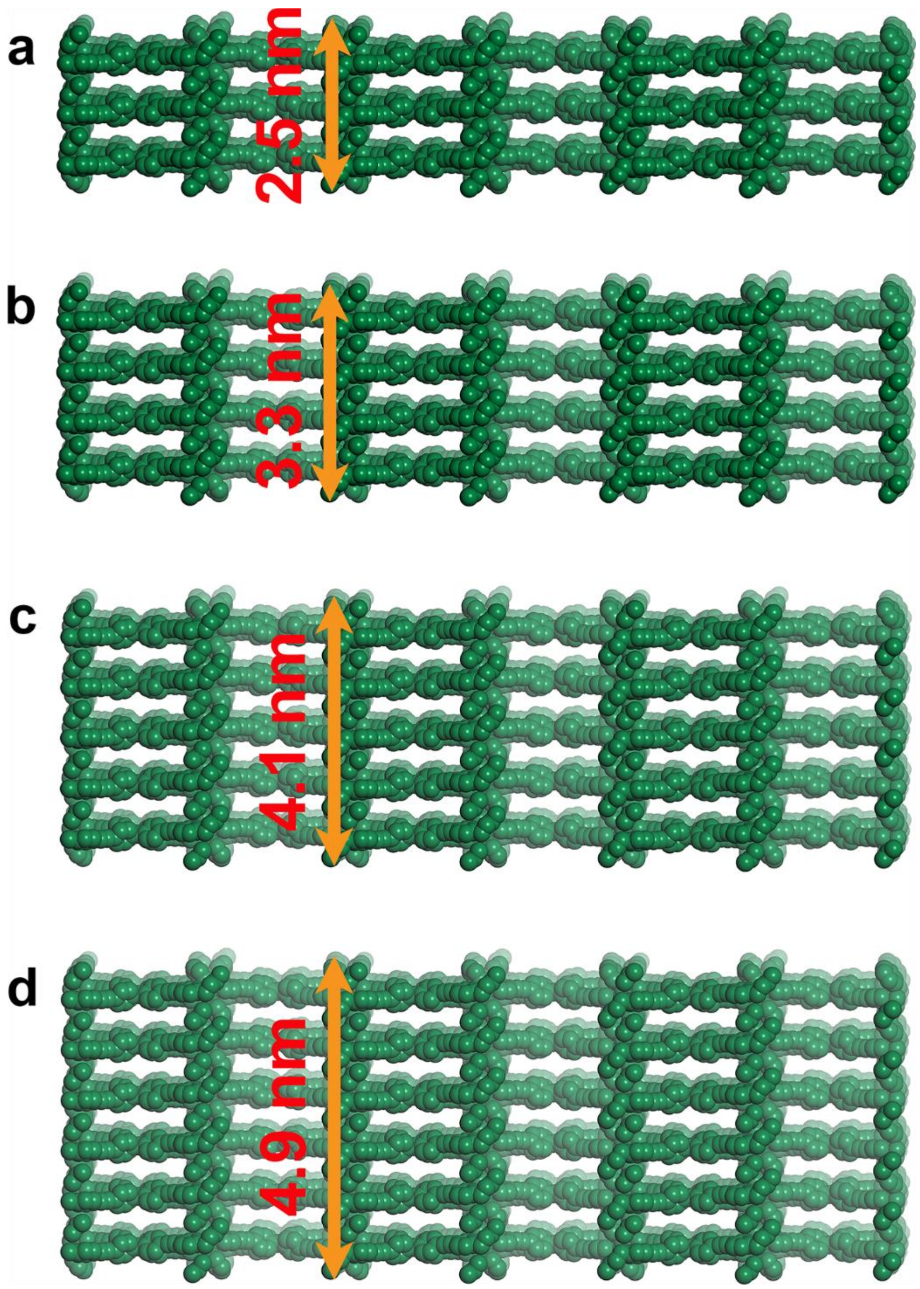

Supplementary Figure 17 | The theoretical height of NUS-24 nanosheets based on the AA stacking model. a, Three-layered (2.5 nm). **b**, Four-layered (3.3 nm). **c**, Five-layered (4.1 nm). **d**, Six-layered (4.9 nm).

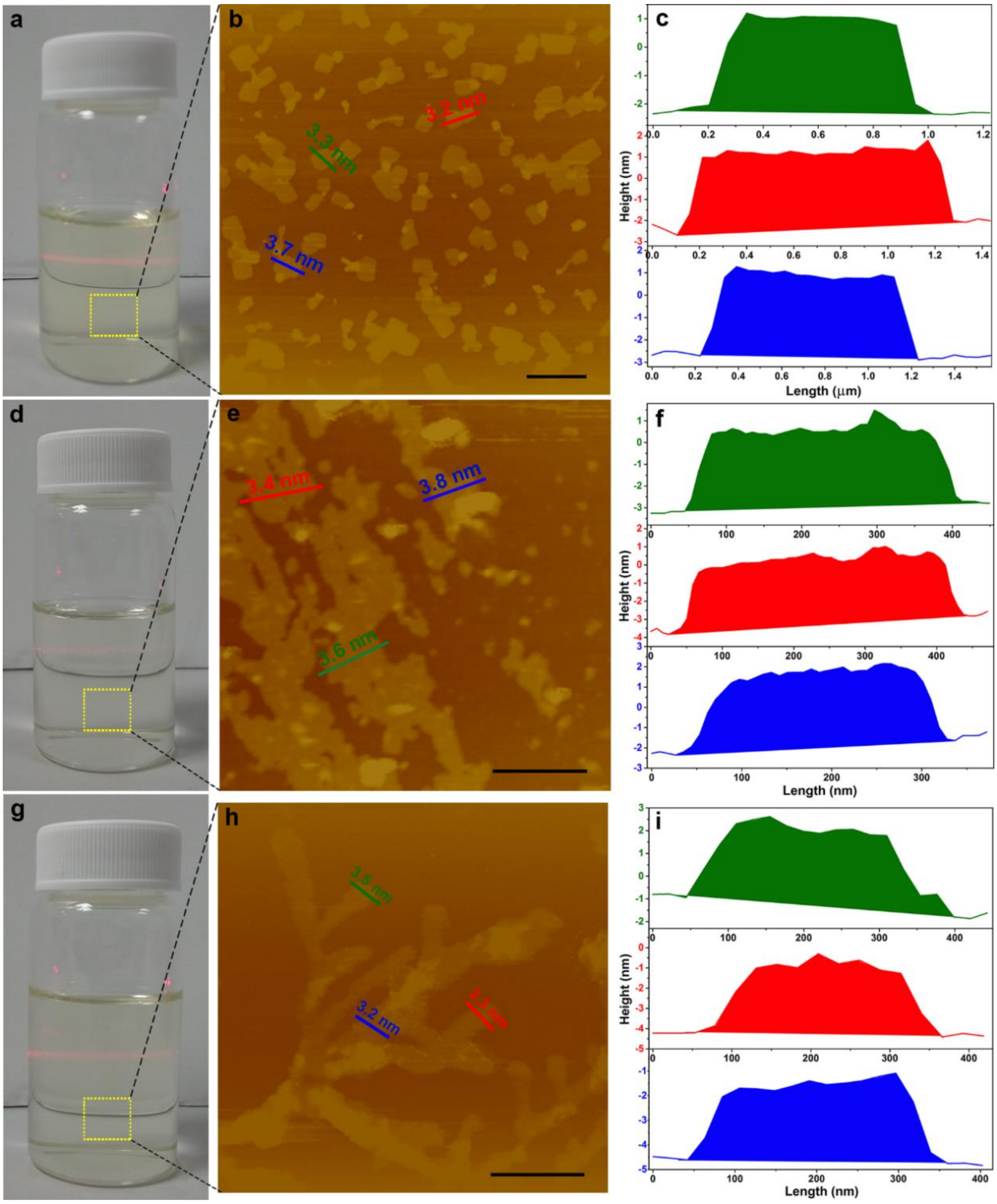

Supplementary Figure 18 | AFM images of NUS-24 nanosheets after 60 days. a-c, The Tyndall effect and AFM images of exfoliated NUS-24 nanosheets in acetone after 60 days (Scale bar, 2 μm in **b**). **d-f**, The Tyndall effect and AFM images of exfoliated NUS-24 nanosheets in acetonitrile after 60 days (Scale bar, 0.5 μm in **e**). **g-i**, The Tyndall effect and AFM images of exfoliated NUS-24 nanosheets in ethanol after 60 days (Scale bar, 1 μm in **h**).

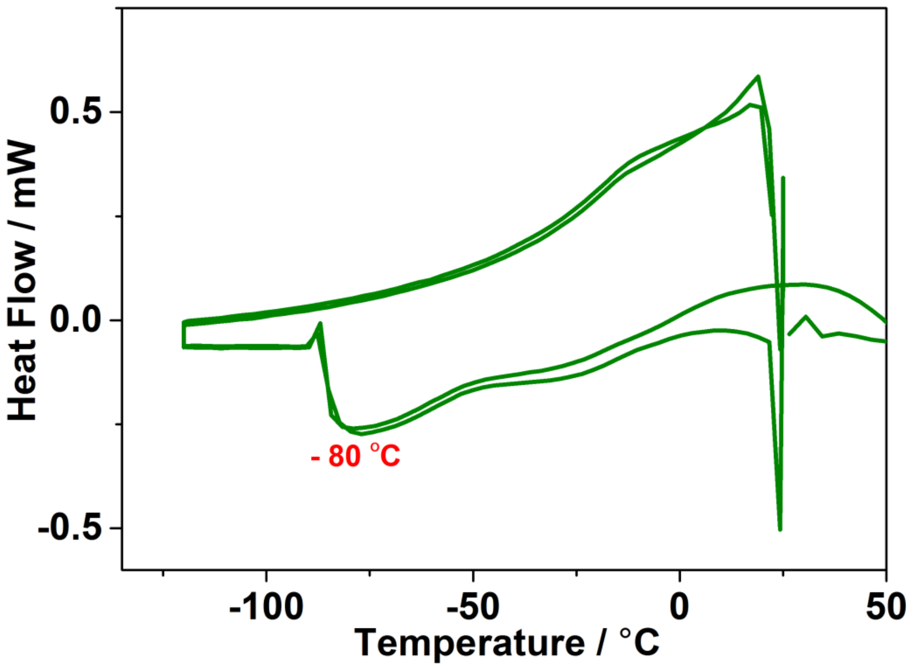

**Supplementary Figure 19 | DSC curve of NUS-24 bulk powder.

.

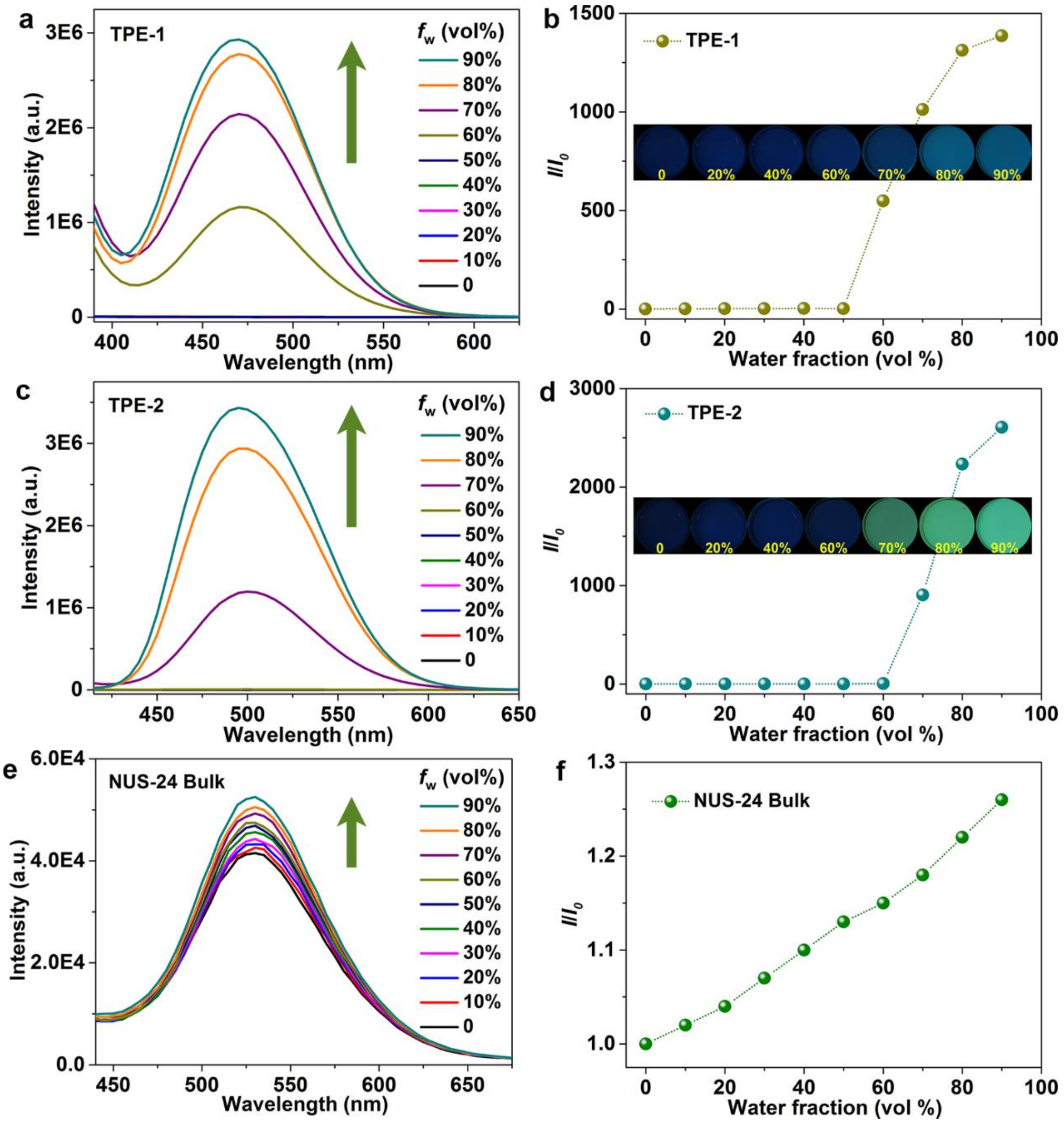

Supplementary Figure 20 | AIE characteristics of TPE-1, TPE-2 and NUS-24 bulk powder. a and **c**, Fluorescent spectra of TPE-1 (c = 1.5 × 10^{-4} M, λ_{ex} = 360 nm) and TPE-2 (c = 5.0 × 10^{-5} M, λ_{ex} = 360 nm) in acetone and acetone/water mixtures. **b** and **d**, Plots of relative emission intensity versus water fraction in acetone/water mixtures of TPE-1 and TPE-2 (Inset: fluorescent photographs with different water fraction). **e**, Fluorescent spectra of NUS-24 bulk powder (c = 0.2 mg mL^{-1}, λ_{ex} = 365 nm) in acetone and acetone/water mixtures. **f**, Plot of relative emission intensity versus water fraction in acetone/water mixtures of NUS-24 bulk powder (I = peak intensity in acetone/water mixtures and I_0 = peak intensity in pure acetone).

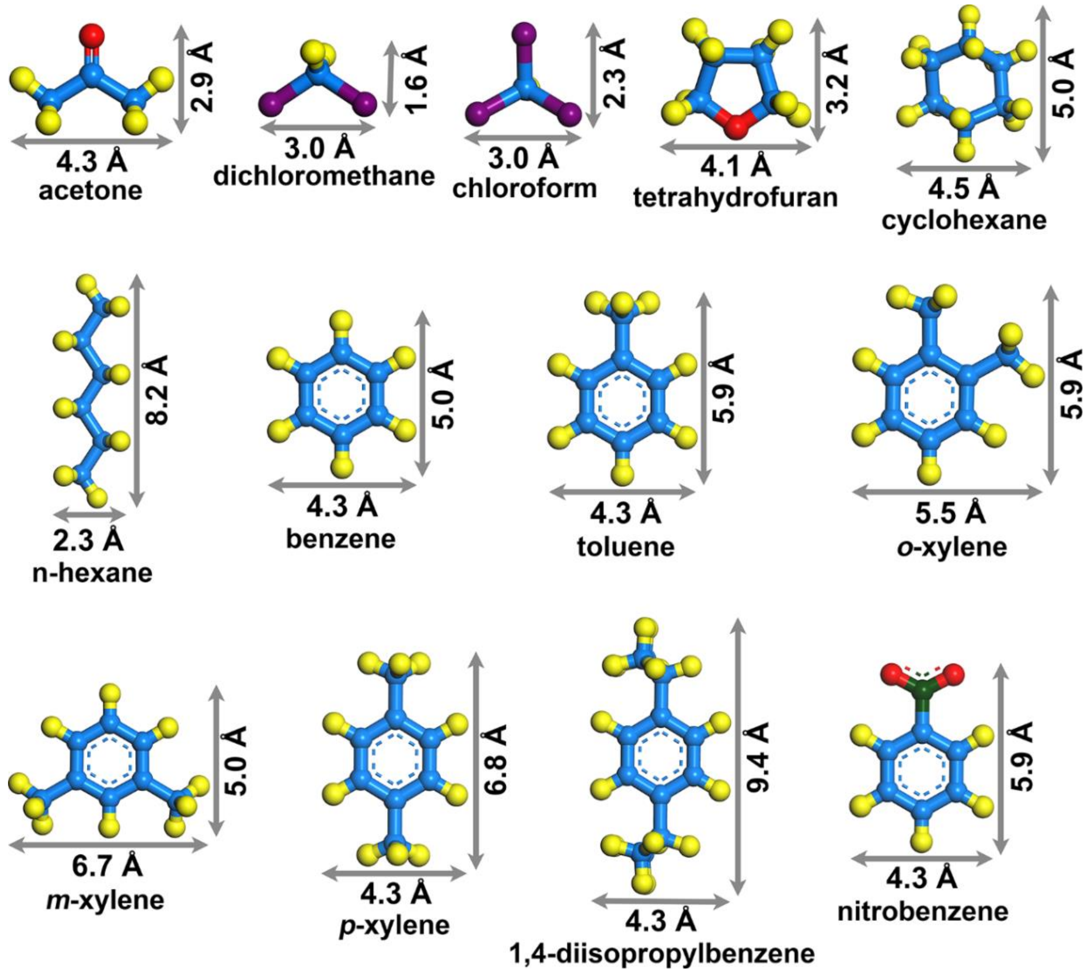

Supplementary Figure 21 | The chemical structure and molecular size of VOCs used in this study.

Supplementary Note 4: DFT calculations were performed on thirteen guest molecules including 1,4-isopropylbenzene, acetone, benzene, dichloromethane, chloroform, cyclohexane, *m*-xylene, *n*-hexane, nitrobenzene, *o*-xylene, *p*-xylene, tetrahydrofuran and toluene. All the guest molecules were optimized using the B3LYP[7] hybrid functional with 6-31G (d) basis set. We have measured the molecular size of these VOCs by “square” dimensions shown in Supplementary Figure 21.

.

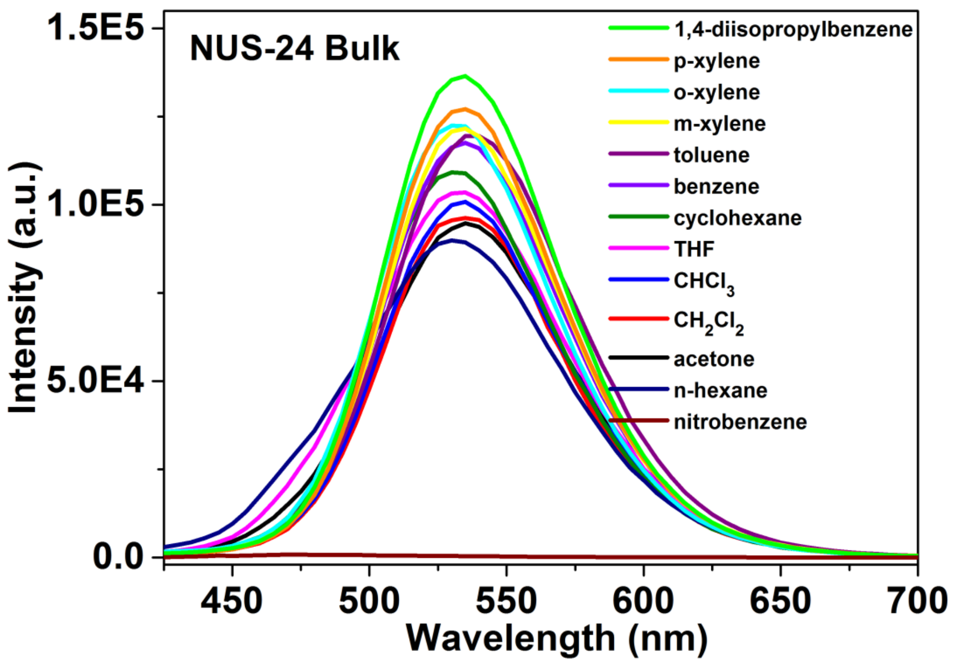

Supplementary Figure 22 | Fluorescence emission spectra of NUS-24 bulk powder suspended in various VOC solutions at room temperature (c = 0.5 mg mL^{-1}, λ_{ex} = 365 nm).

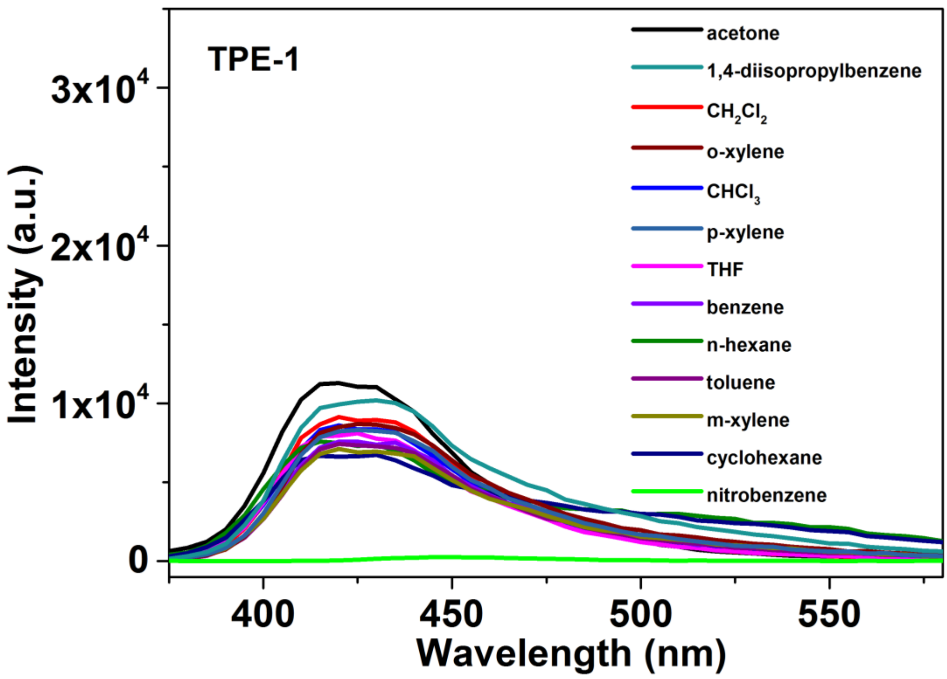

Supplementary Figure 23 | Fluorescence emission spectra of TPE-1 linker in different VOCs at room temperature (c = 0.25 mg mL^{-1}, λ_{ex} = 365 nm).

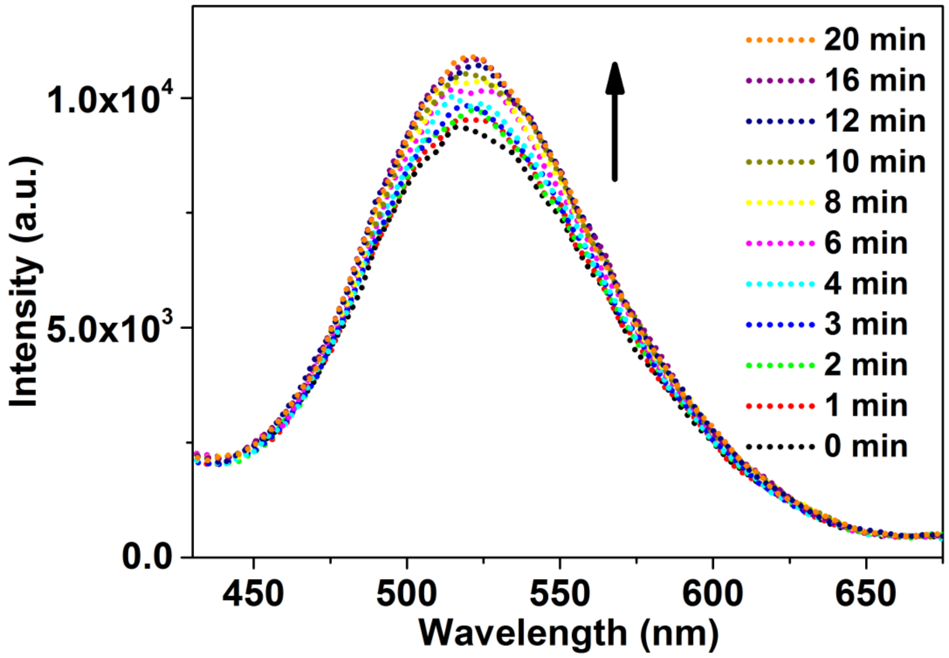

Supplementary Figure 24 | Time-dependent emission spectra of NUS-24 nanosheets in 1,4-diisopropylbenzene (20% v/v in acetone, λ_{ex} = 365 nm).

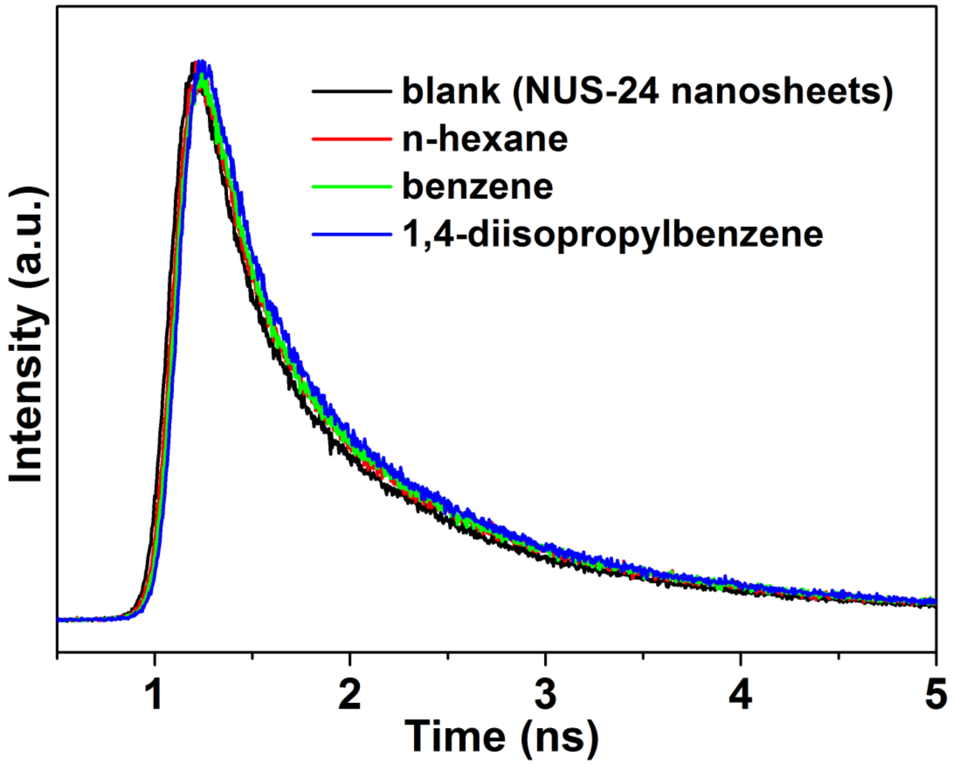

Supplementary Figure 25 | Emission decay trace of NUS-24 nanosheets in acetone (blank), *n*-hexane, benzene and 1,4-diisopropylbenzene.

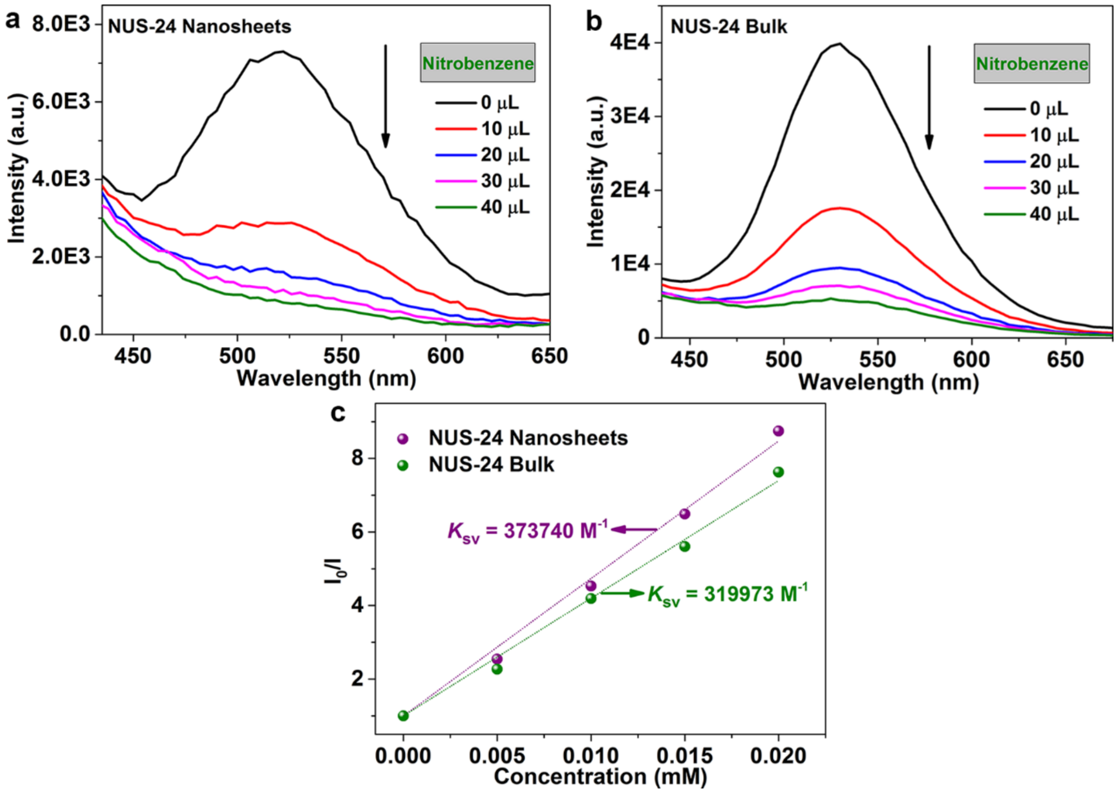

Supplementary Figure 26 | Fluorescence titration of NUS-24 nanosheets and NUS-24 bulk powder by nitrobenzene. a, Fluorescence emission spectra of NUS-24 nanosheets (c = 20 μg mL^{-1}) upon titration with nitrobenzene solution (1×10^{-3} M) at room temperature (λ_{ex} = 365 nm). **b**, Fluorescence emission spectra of NUS-24 bulk (c = 0.2 mg mL^{-1}) upon titration with nitrobenzene solution (1×10^{-3} M) at room temperature (λ_{ex} = 365 nm). **c**, Stern−Völmer plots of NUS-24 nanosheets and NUS-24 bulk powder being titrated with nitrobenzene.

Supplementary Note 5: Fluorescence titration was performed by gradually adding trace amounts of nitrobenzene into the acetone suspensions containing either NUS-24 nanosheets or NUS-24 bulk powder. For NUS-24 nanosheets, the measured I_0/I varies linearly with nitrobenzene concentration (R^2 > 0.99), and the quenching constant K_{SV} was calculated to be 373740 M^{-1}. In addition, we have noticed that the fluorescence of NUS-24 nanosheets can be quenched faster than that of the bulk powder [K_{sv} constant: 373740 M^{-1} (nanosheets) vs 319973 M^{-1} (bulk)], suggesting a higher sensitivity of nanosheets even under quenching mode.

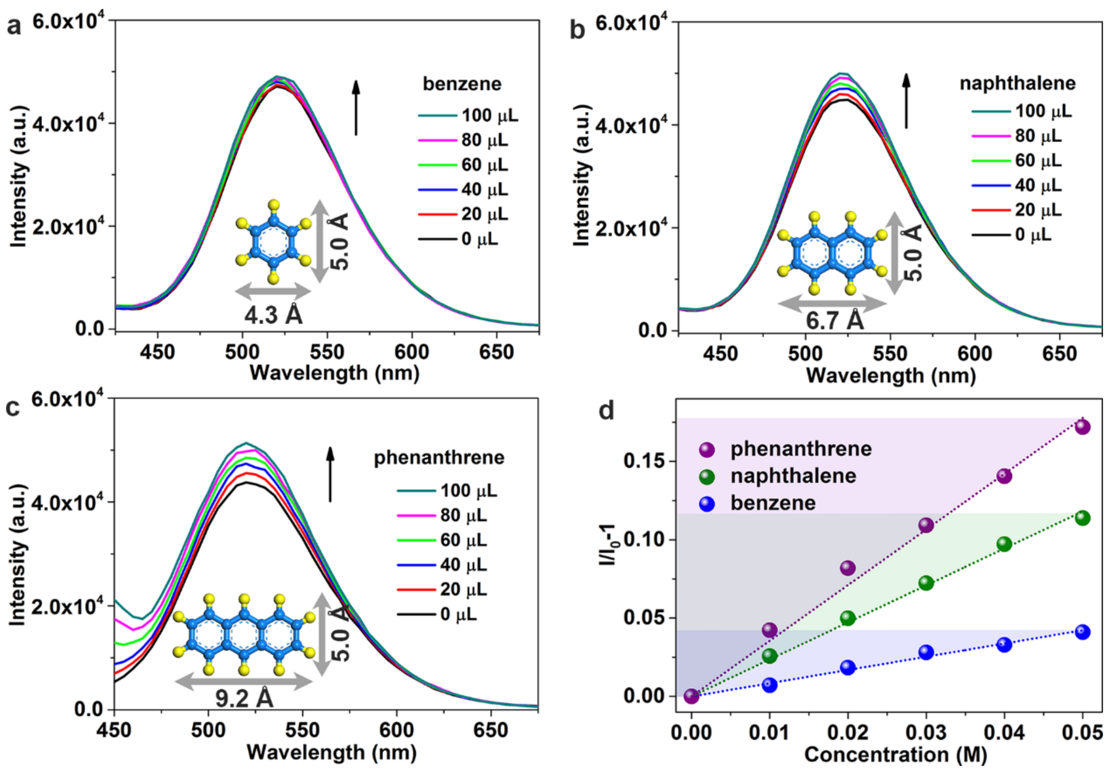

Supplementary Figure 27 | Fluorescence titration of NUS-24 nanosheets by analytes with different size. a, Fluorescence emission spectra of NUS-24 nanosheets (50 μg mL^{-1} in acetone, λ_{ex} = 365 nm) upon titration with benzene (1 M). **b**, Fluorescence emission spectra of NUS-24 nanosheets (50 μg mL^{-1} in acetone, λ_{ex} = 365 nm) upon titration with naphthalene (1 M). **c**, Fluorescence emission spectra of NUS-24 nanosheets (50 μg mL^{-1} in acetone, λ_{ex} = 365 nm) upon titration with phenanthrene (1 M). **d**, Fluorescence emission intensity versus analyte concentration plots of NUS-24 nanosheets titrated with benzene, naphthalene and phenanthrene, respectively.

Supplementary Note 6: The measured absorbance I/I_0-1 (I_0 is the original peak maximum intensity of the nanosheets and I is the peak maximum intensity after exposure to these analytes) at 520 nm varies as a function of analyte concentration [M] in linear relationships (R^2 > 0.99).

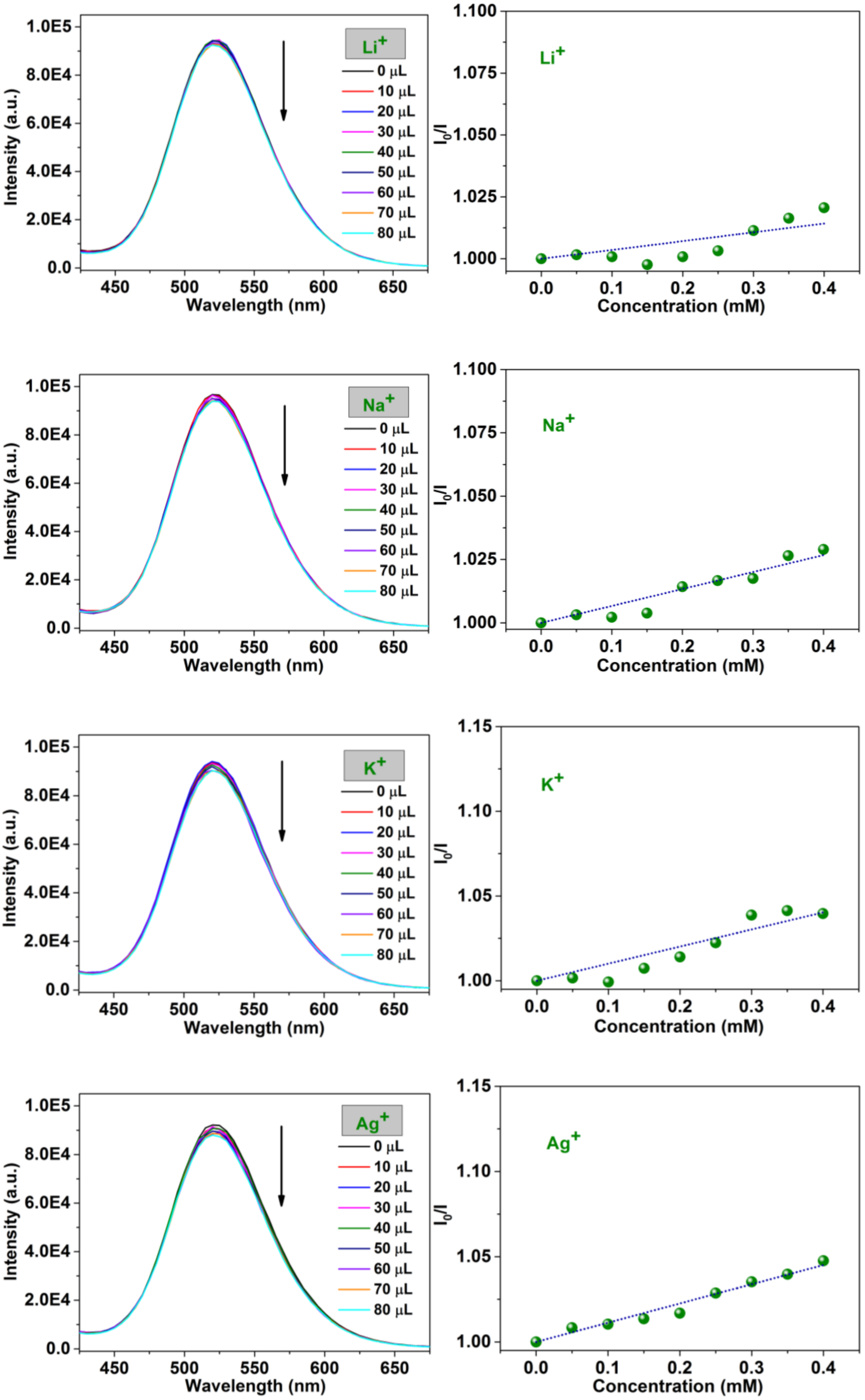
Li+
0 μL
10 μL
20 μL
30 μL
40 μL
50 μL
60 μL
70 μL
80 μL
Intensity (a.u.)
Wavelength (nm)
I0/I
Concentration (mM)
Na+
K+
Ag+

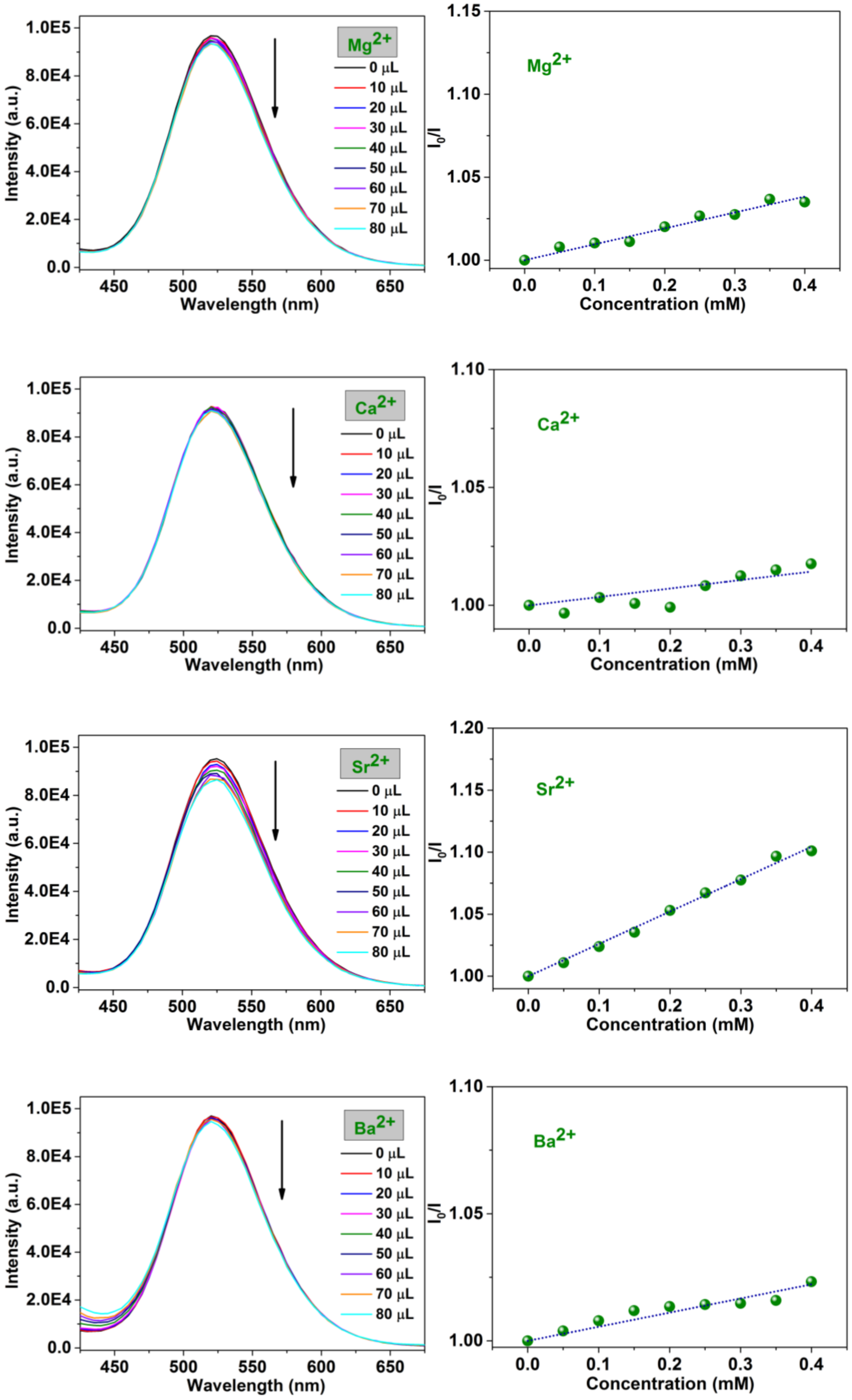
Mg2+
0 μL
10 μL
20 μL
30 μL
40 μL
50 μL
60 μL
70 μL
80 μL
Intensity (a.u.)
Wavelength (nm)
I0/I
Concentration (mM)
Ca2+
Sr2+
Ba2+

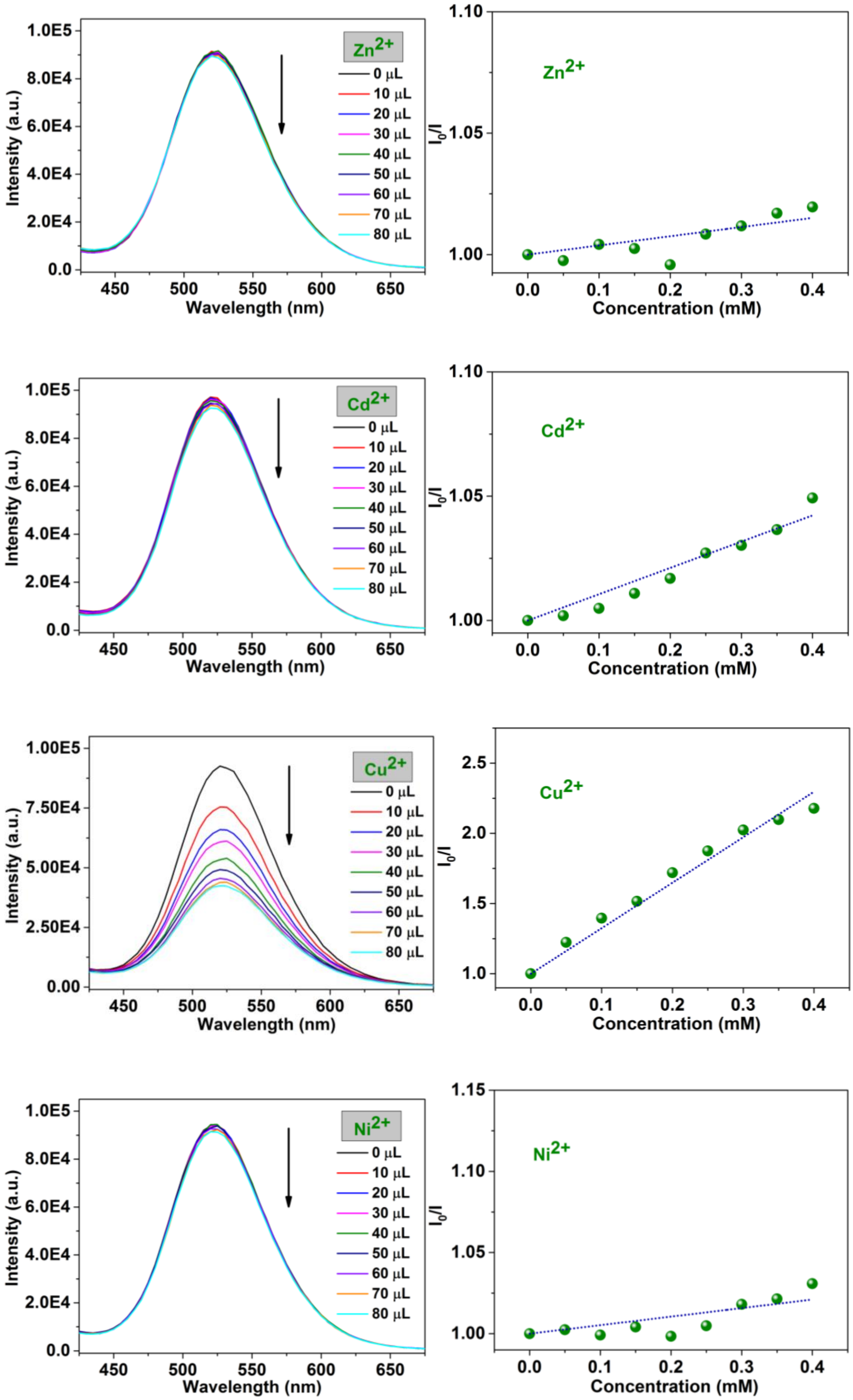
Zn2+
0 μL
10 μL
20 μL
30 μL
40 μL
50 μL
60 μL
70 μL
80 μL
Intensity (a.u.)
Wavelength (nm)
I0/I
Concentration (mM)
Cd2+
Cu2+
Ni2+

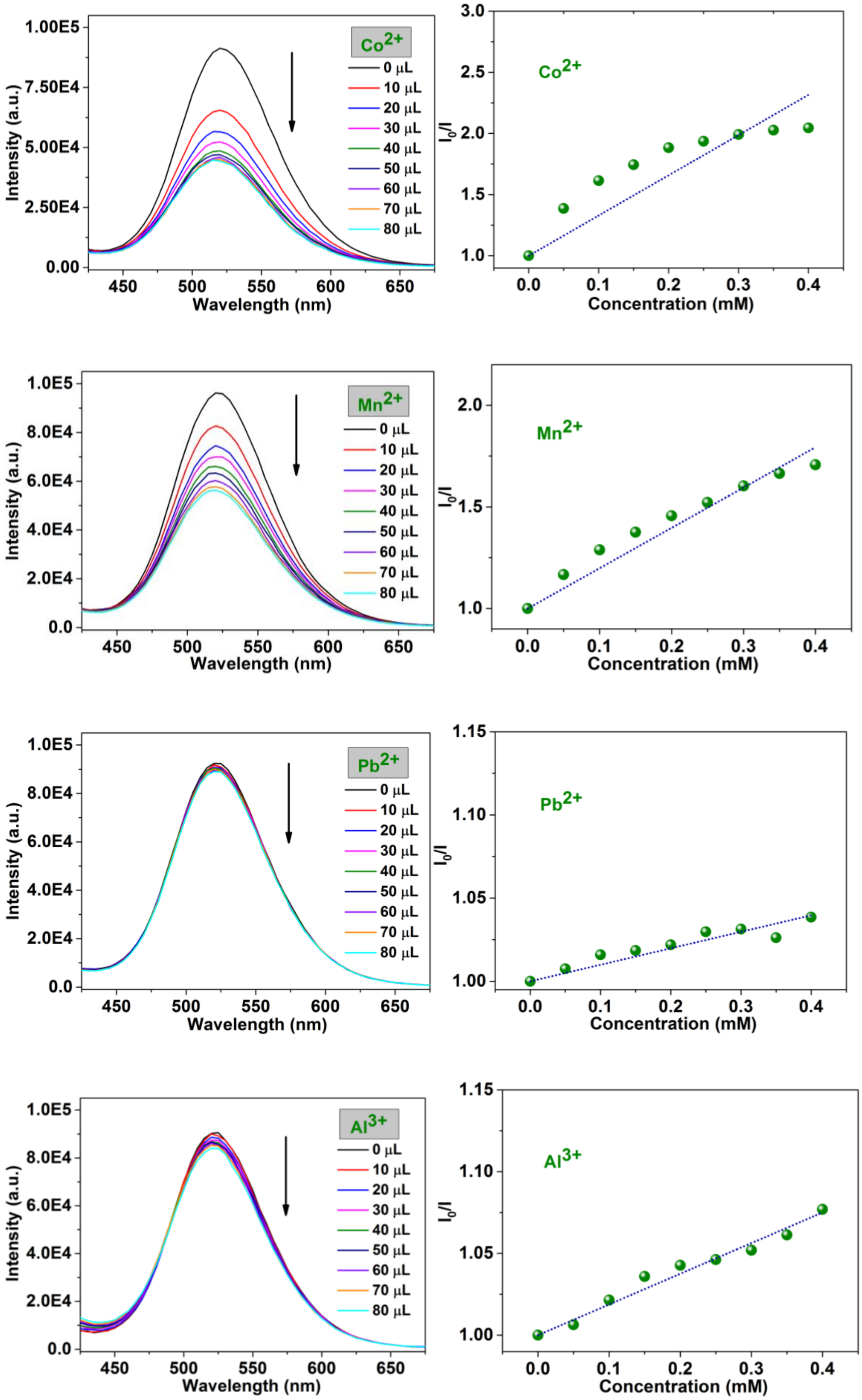
Co2+
0 μL
10 μL
20 μL
30 μL
40 μL
50 μL
60 μL
70 μL
80 μL
Intensity (a.u.)
Wavelength (nm)
I0/I
Concentration (mM)
Mn2+
Pb2+
Al3+

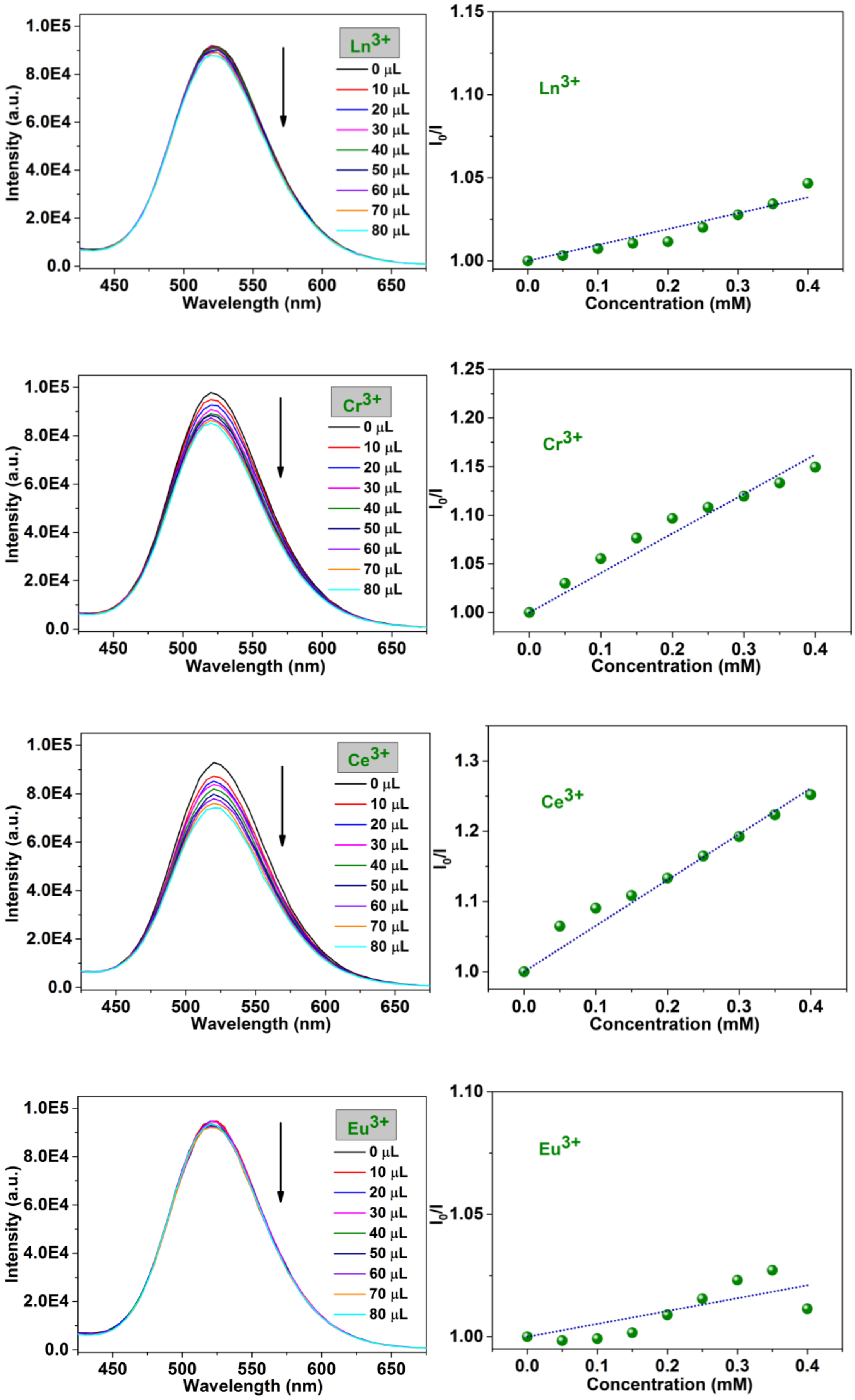

Intensity (a.u.)
Wavelength (nm)
Ln3+
Cr3+
Ce3+
Eu3+
0 μL
10 μL
20 μL
30 μL
40 μL
50 μL
60 μL
70 μL
80 μL
I0/I
Concentration (mM)

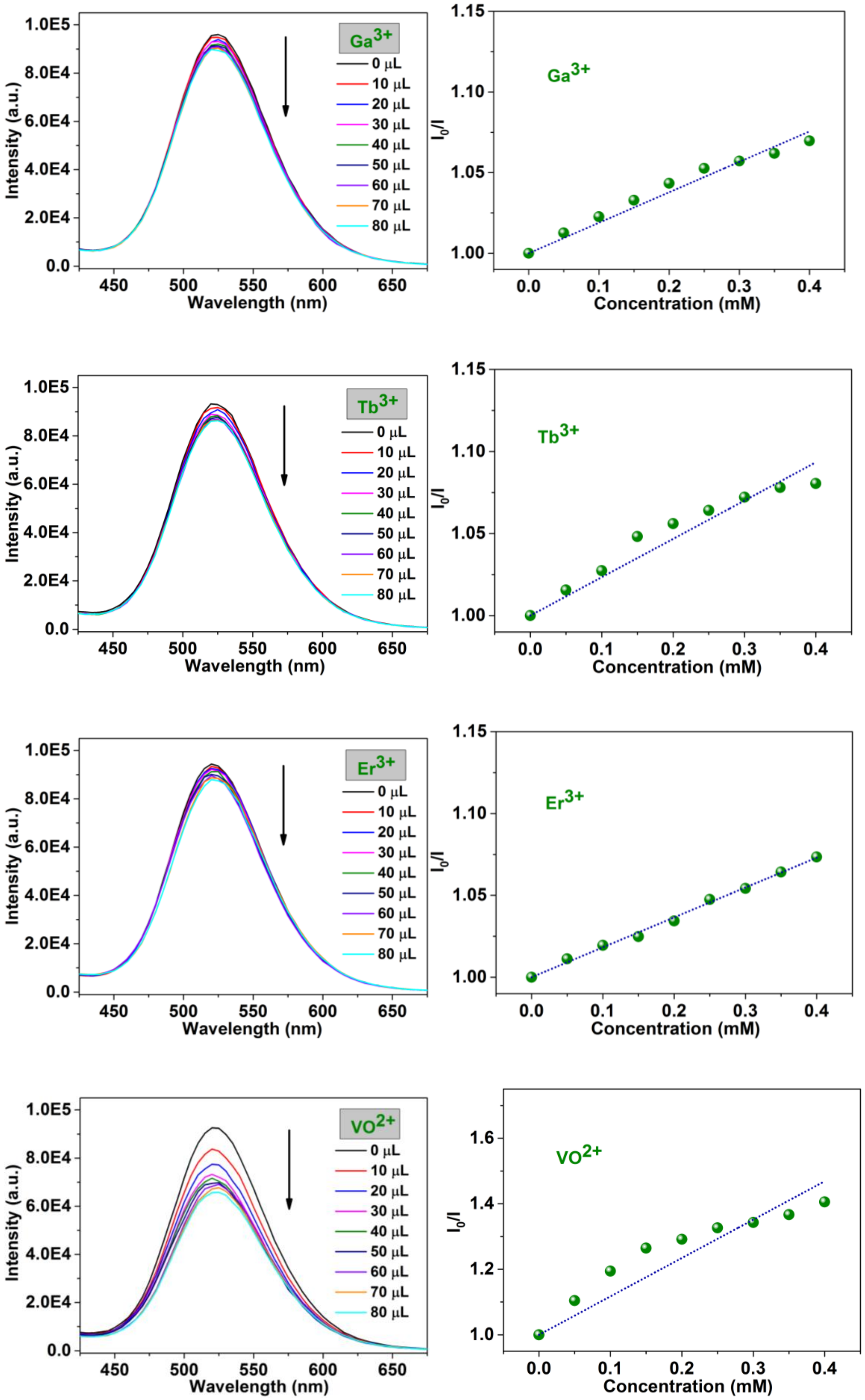

Ga3+
Tb3+
Er3+
VO2+
0 μL
10 μL
20 μL
30 μL
40 μL
50 μL
60 μL
70 μL
80 μL
Intensity (a.u.)
Wavelength (nm)
I0/I
Concentration (mM)

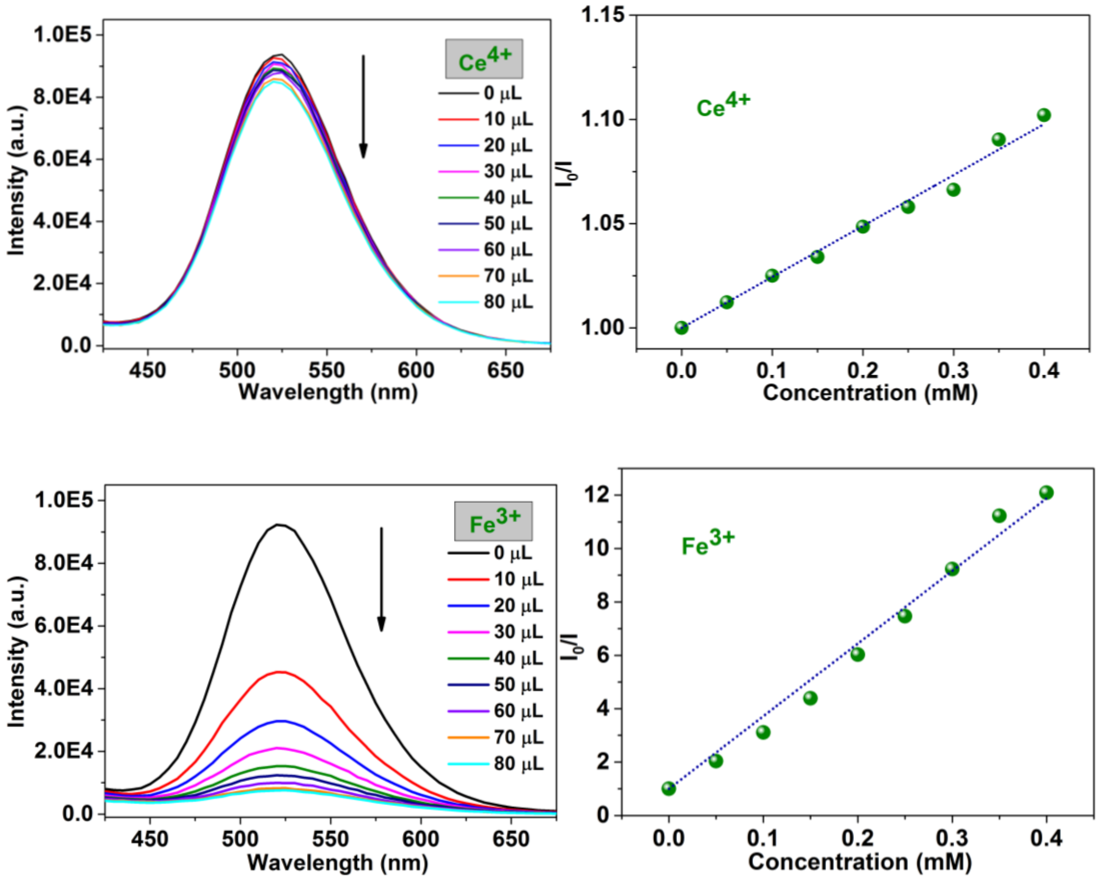

Supplementary Figure 28 | Fluorescence emission spectra of NUS-24 nanosheets (*c* = 0.1 mg mL^{-1}) upon titration with different metal ion solutions (1 × 10^{-2} M) at room temperature (λ_{ex} = 365 nm).

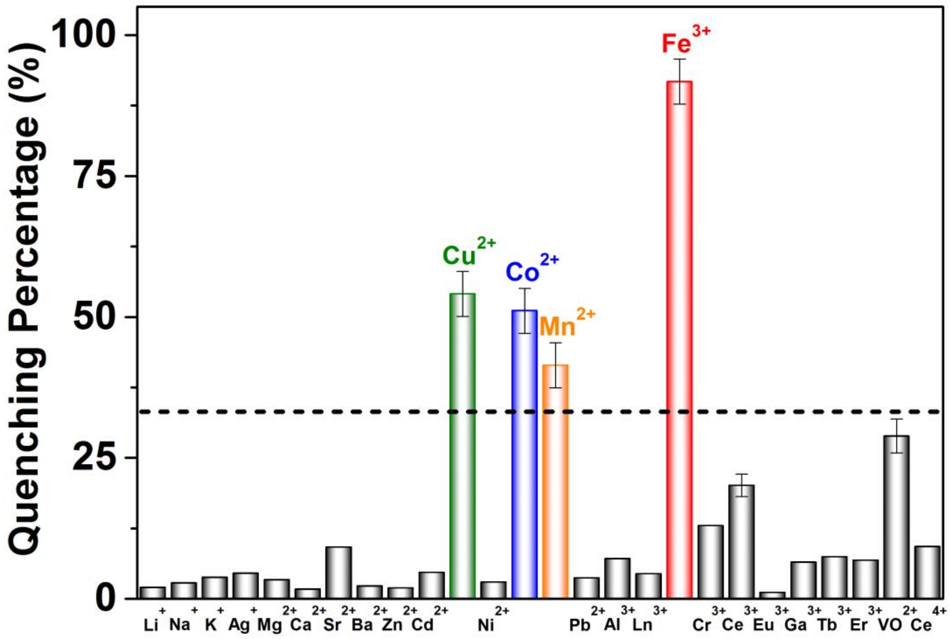

Supplementary Figure 29 | The quenching percentages of NUS-24 nanosheets by different metal ions (1×10^{-2} M). The quenching percentage (%) was estimated using the formula $(I_0\text{-}I)/I_0 \times 100\%$, where I_0 is the maximum fluorescence intensity of NUS-24 nanosheets before titration with metal ions, I is the maximum intensity after titration with metal ions.

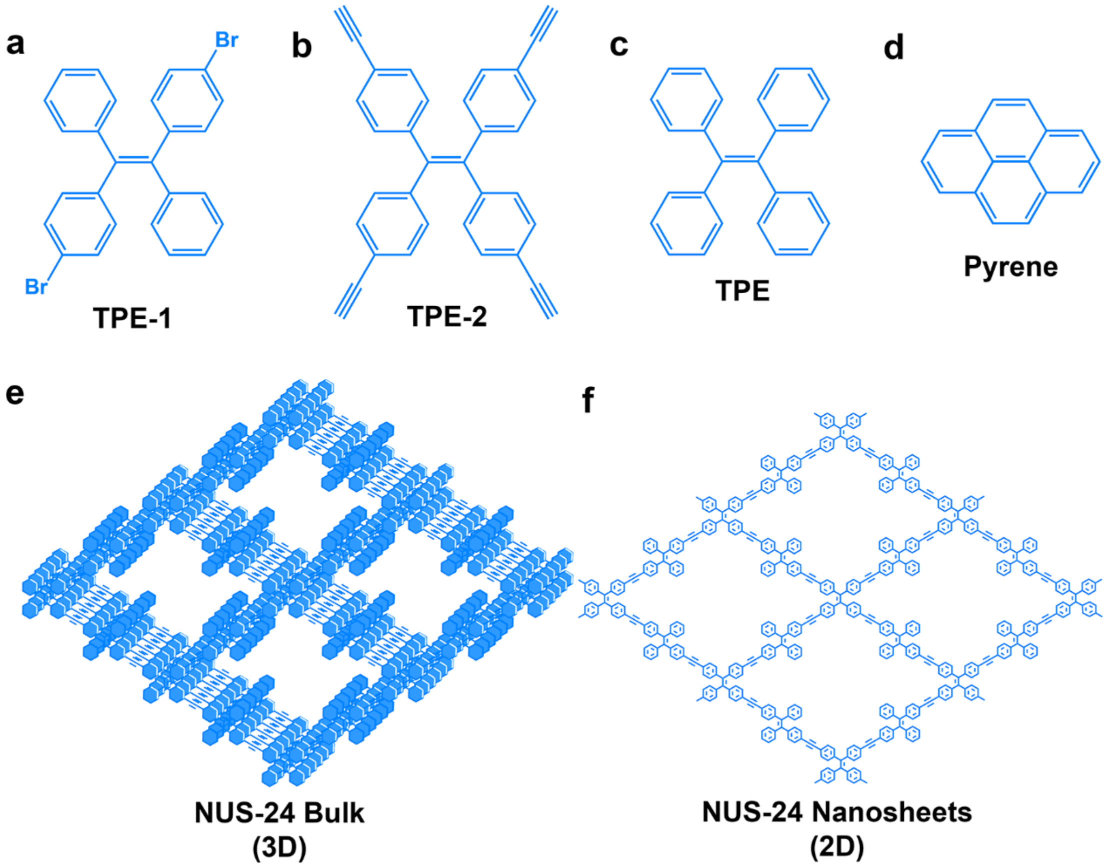

Supplementary Figure 30 | The molecular structures of molecules and materials used in control experiment for metal ion sensing. **a**, TPE-1. **b**, TPE-2. **c**, TPE. **d**, pyrene. **e**, The schematic structure of NUS-24 bulk powder. **f**, The schematic structure of NUS-24 nanosheets.

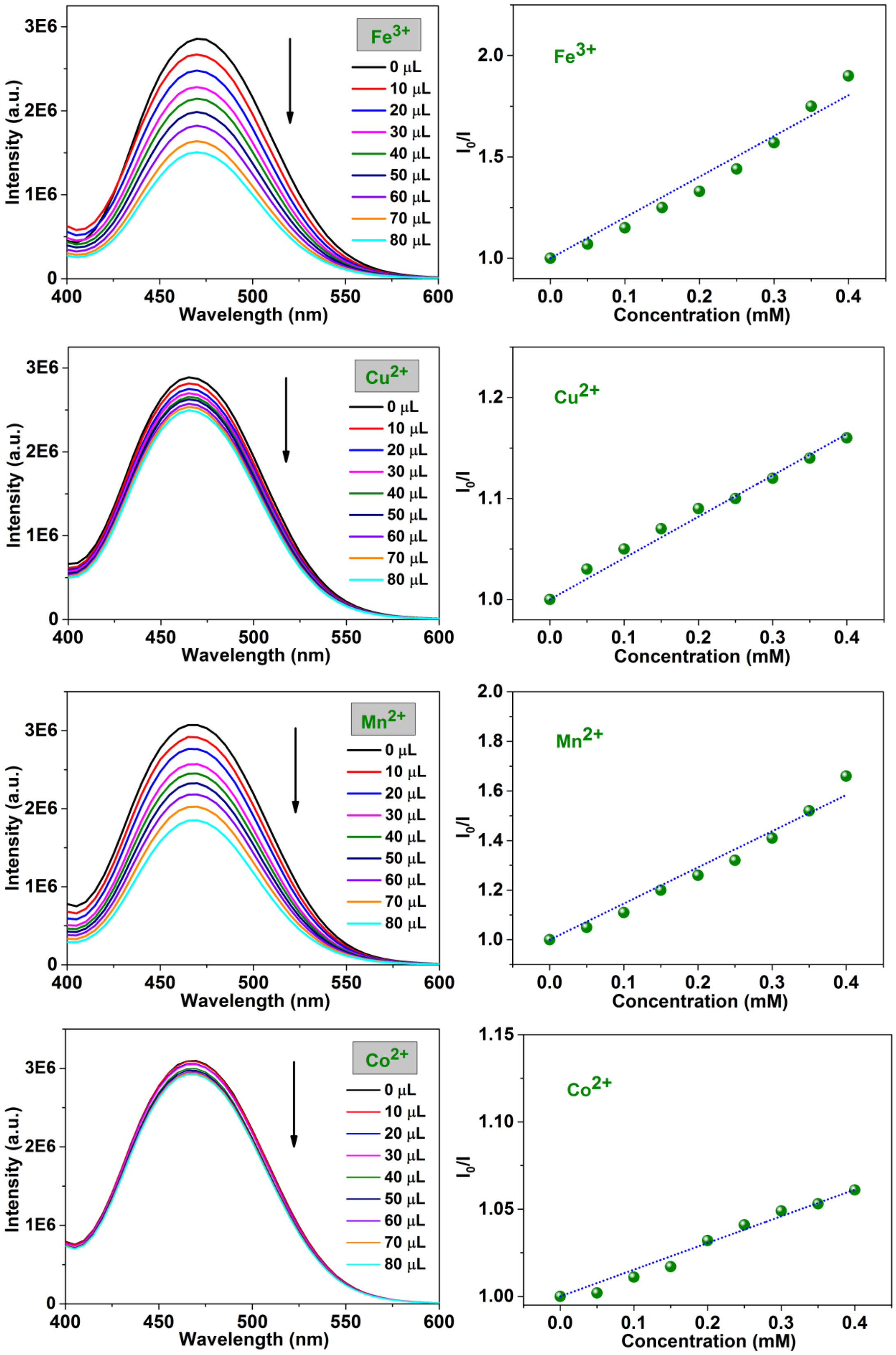

Supplementary Figure 31 | Fluorescence emission spectra of TPE-1 (c = 1.5 $\times 10^{-4}$ M; water/acetone, 90:10, v:v) upon titration with metal ion solutions (1 $\times 10^{-2}$ M) at room temperature (λ_{ex} = 360 nm).

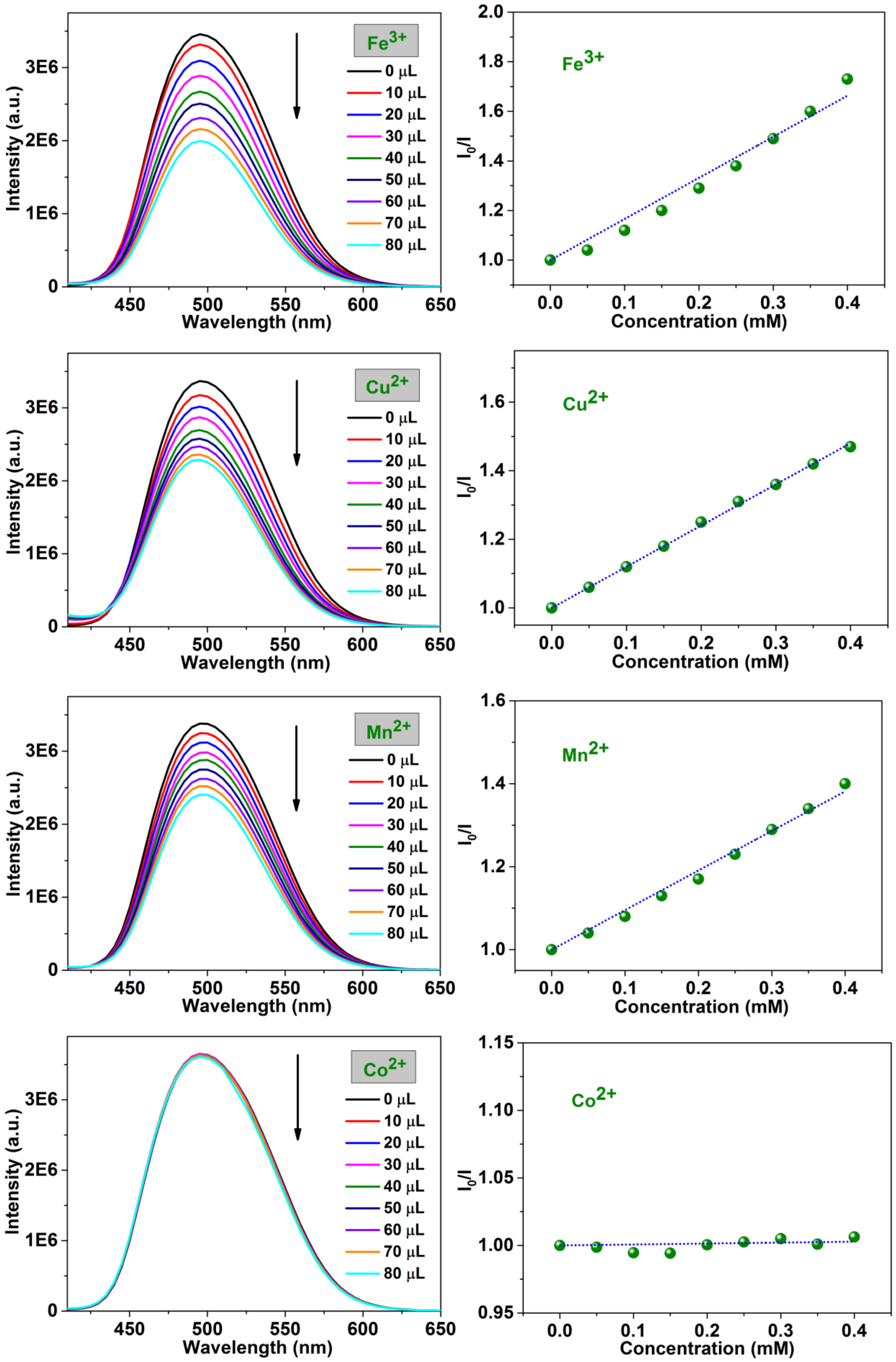

**Supplementary Figure 32 | ** Fluorescence emission spectra of TPE-2 ($c = 5 \times 10^{-5}$ M; water/acetone, 90:10, v:v) upon titration with metal ion solutions (1×10^{-2} M) at room temperature (λ_{ex} = 360 nm).

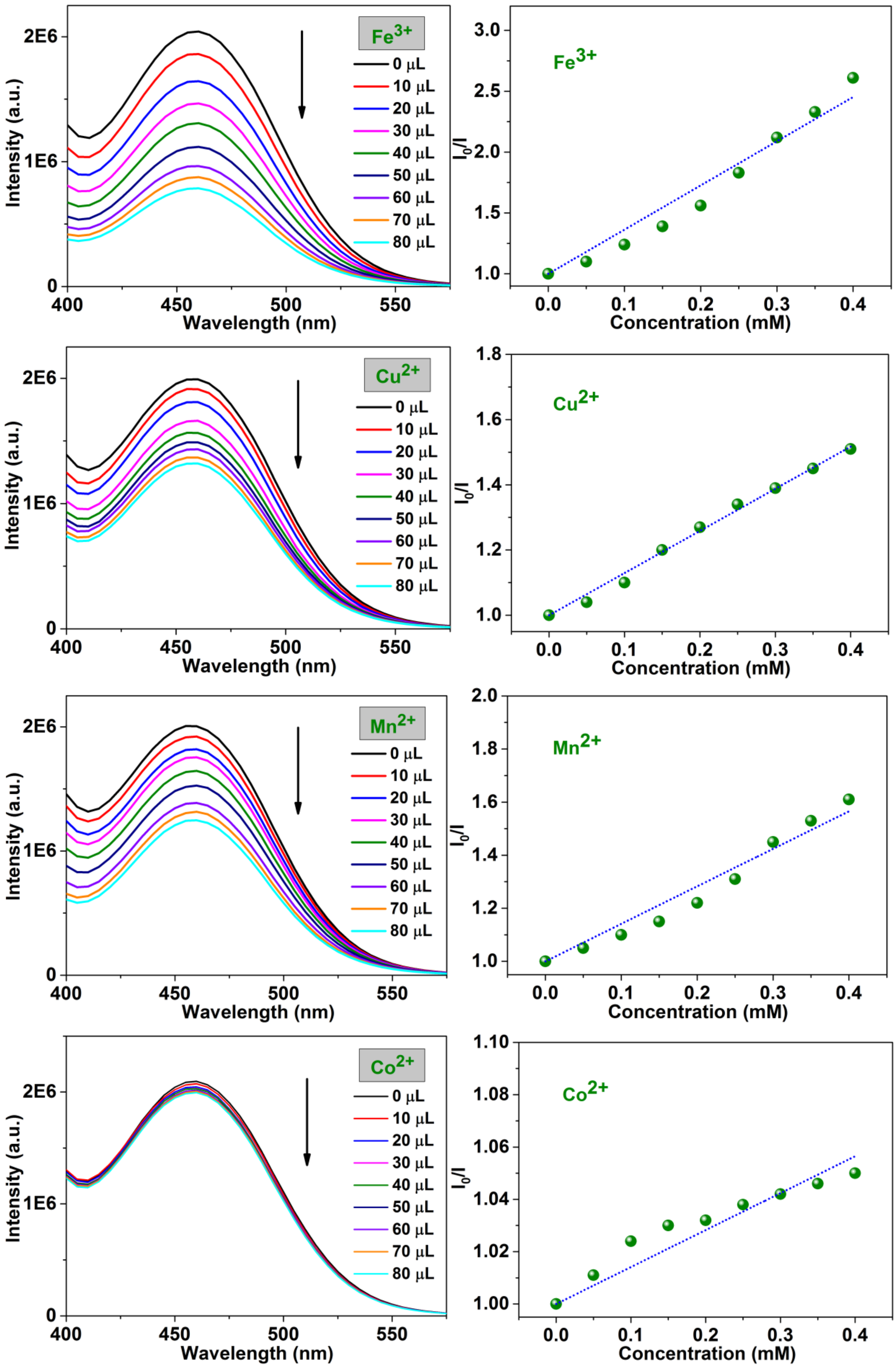

Supplementary Figure 33 | Fluorescence emission spectra of TPE (c = 1.5 × 10^{-4} M; water/acetone, 90:10, v:v) upon titration with metal ion solutions (1 × 10^{-2} M) at room temperature (λ_{ex} = 350 nm).

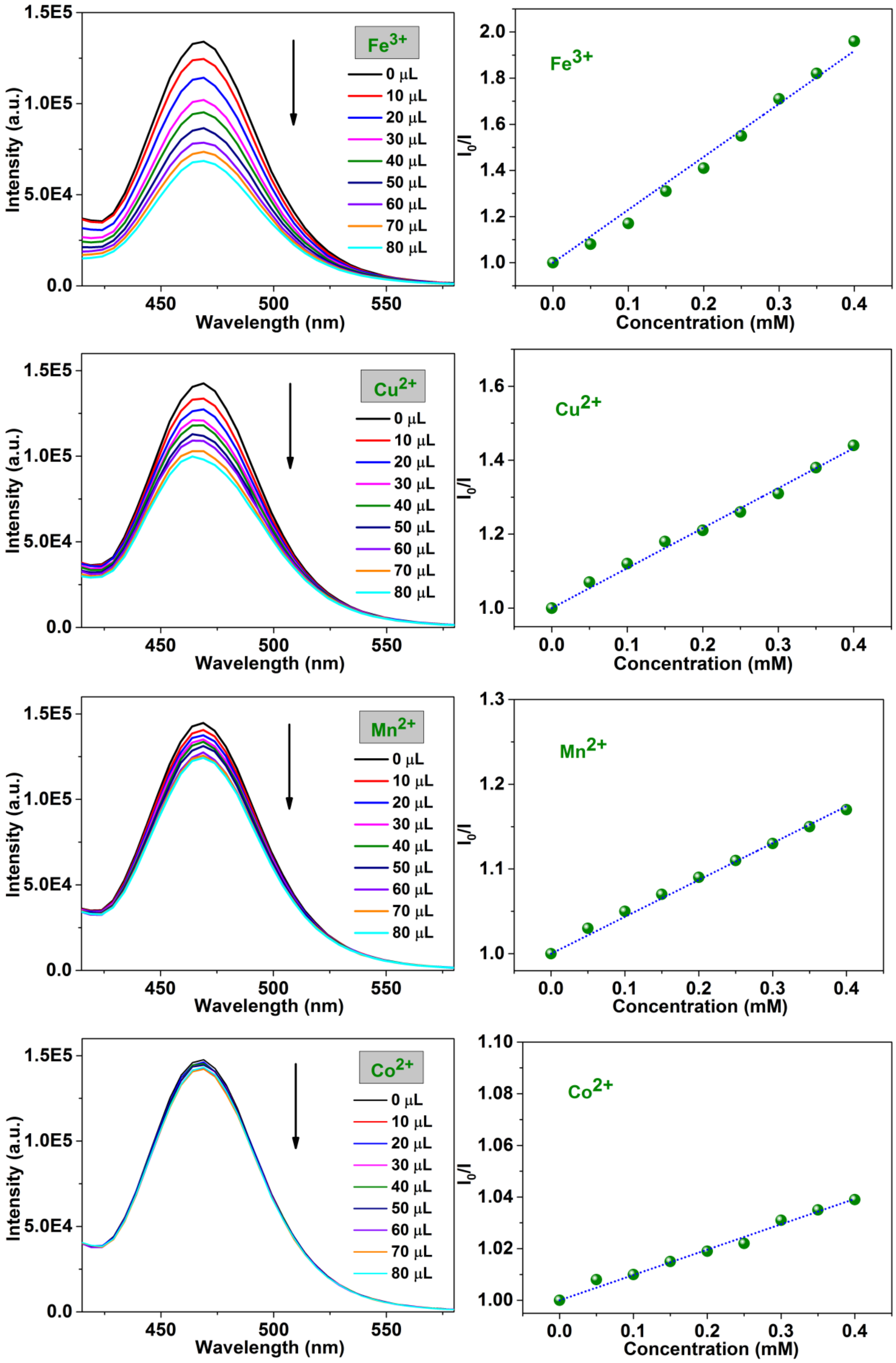

Supplementary Figure 34 | Fluorescence emission spectra of pyrene (c = 7.5 × 10^{-3} M; acetone) upon titration with metal ion solutions (1 × 10^{-2} M) at room temperature (λ_{ex} = 350 nm).

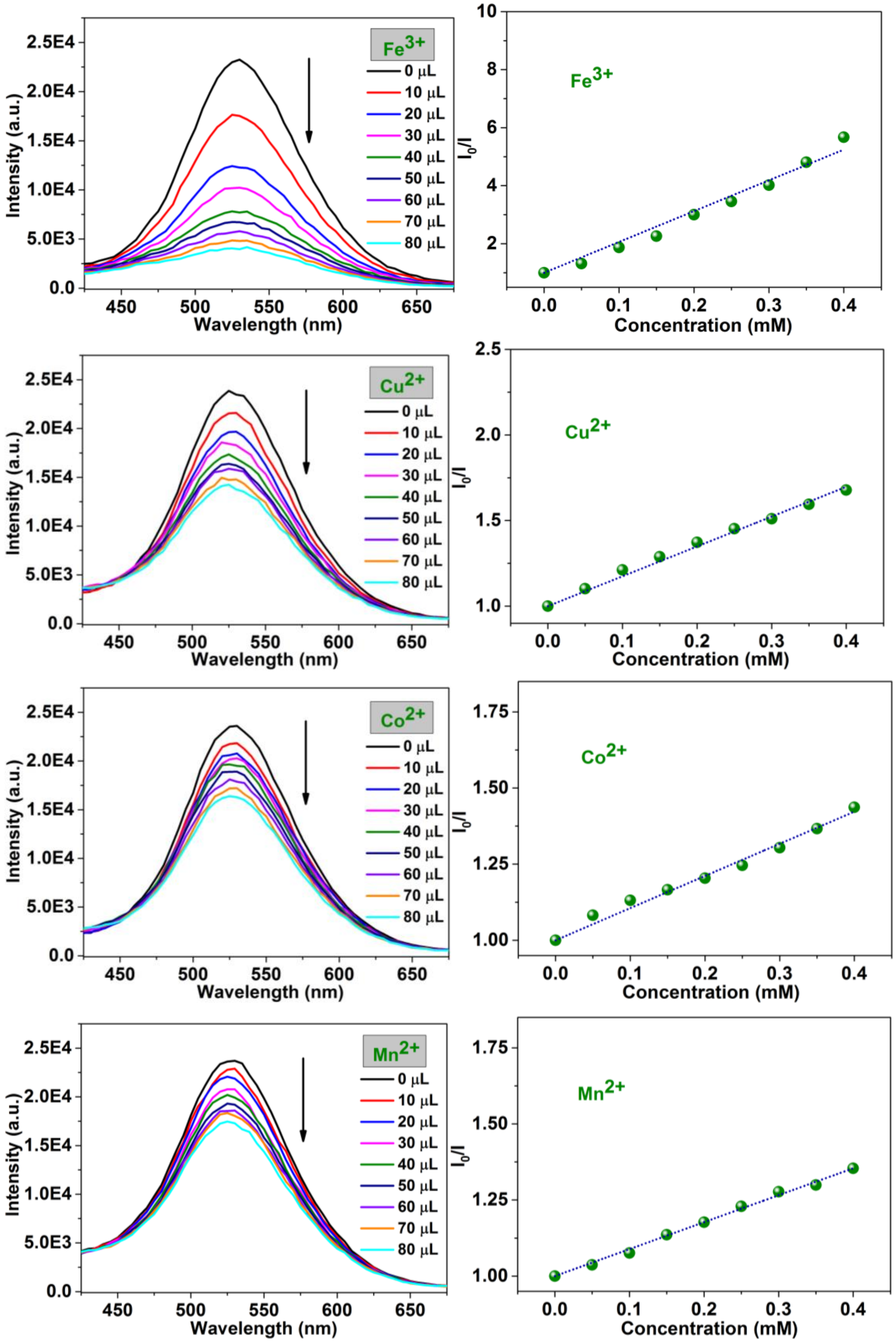

**Supplementary Figure 35 | ** Fluorescence emission spectra of NUS-24 bulk powder (c = 0.1 mg mL^{-1}) upon titration with metal ion solutions (1 × 10^{-2} M) at room temperature (λ_{ex} = 365 nm).

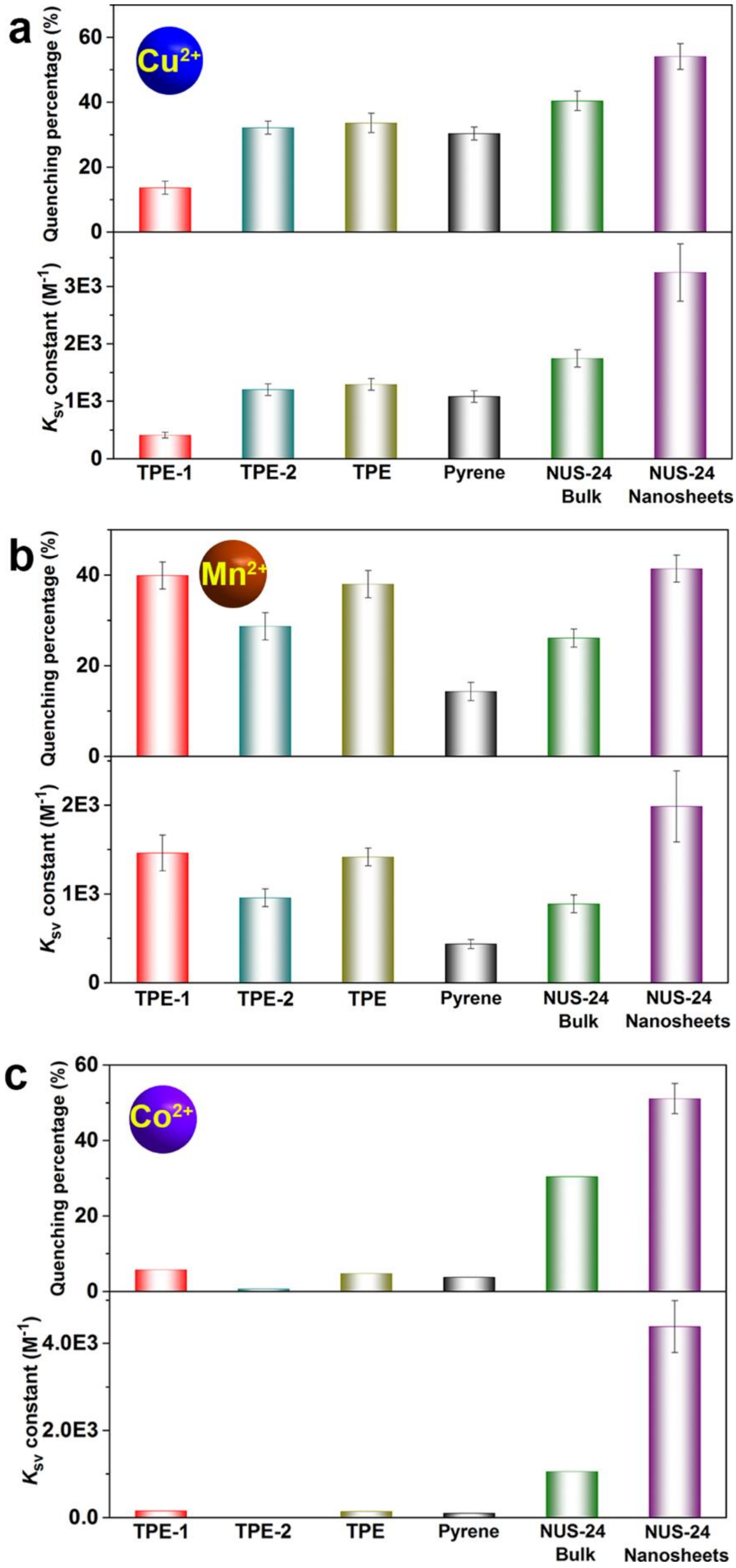

Supplementary Figure 36 | The quenching percentages and K_{SV} constants of TPE-1, TPE-2, TPE, pyrene, NUS-24 bulk powder, and NUS-24 nanosheets by Cu^{2+} (**a**), Mn^{2+} (**b**), and Co^{2+} (**c**).

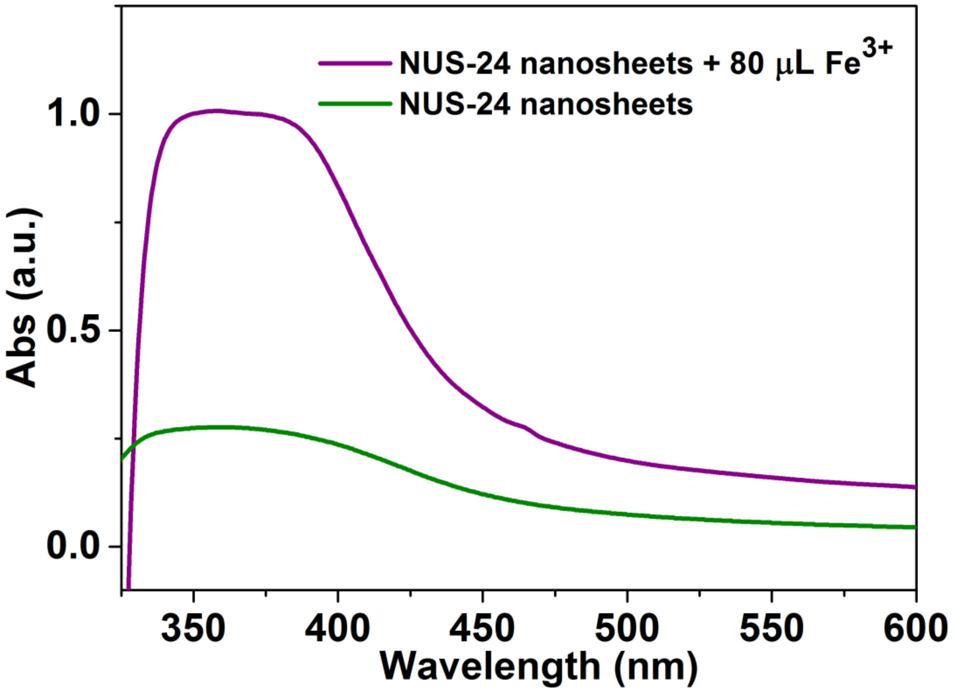

**Supplementary Figure 37 | ** UV−Vis spectra of NUS-24 nanosheets before and after titration with Fe^{3+} solution.

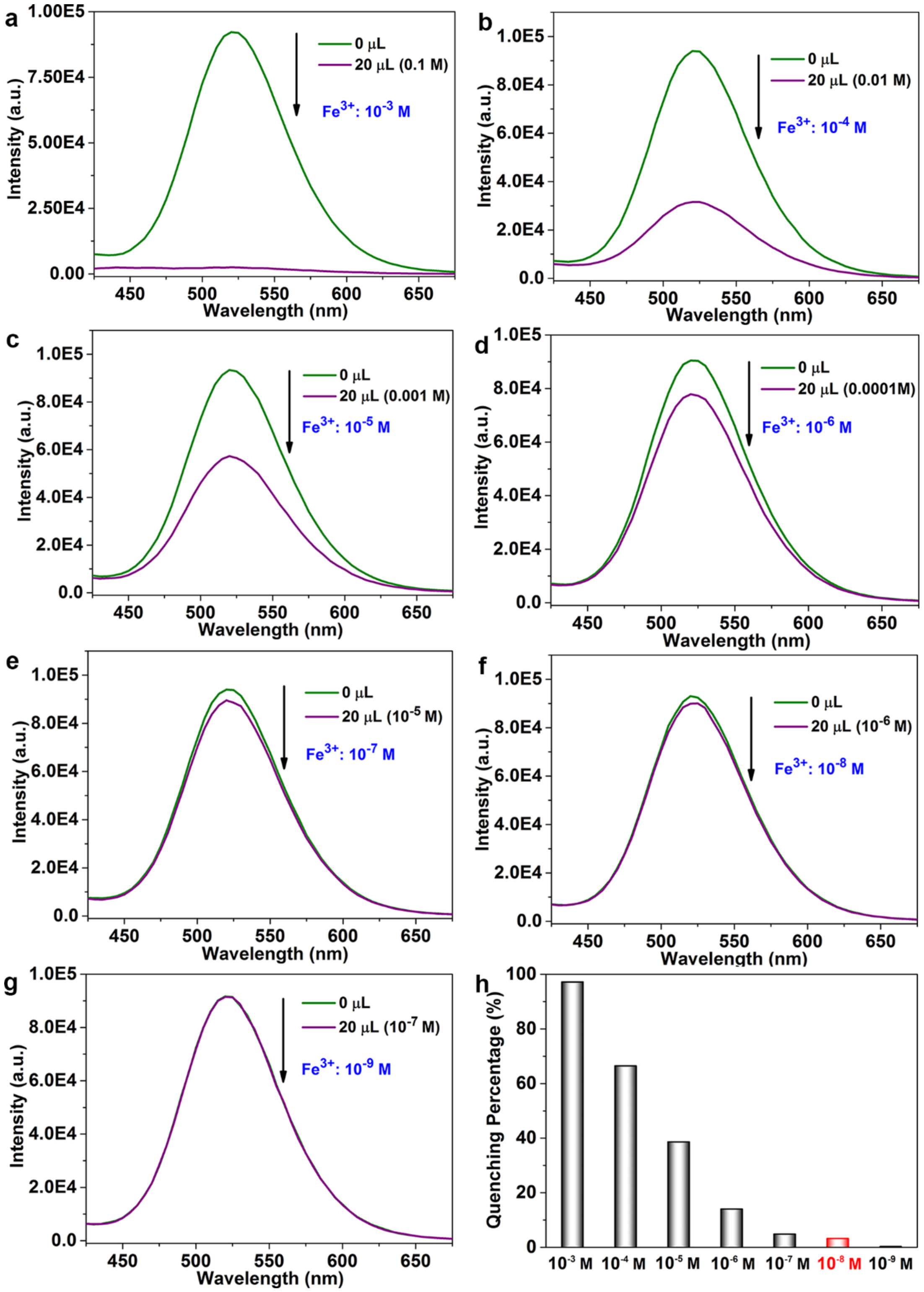

Supplementary Figure 38 | Sensitivity test for the chemical sensing of Fe^{3+} by NUS-24 nanosheets. a-g, Fluorescence emission spectra of NUS-24 nanosheets titrated with Fe^{3+} solution of different concentrations (λ_{ex} = 365 nm). **h**, The quenching percentages of NUS-24 nanosheets by Fe^{3+} solution with different concentrations.

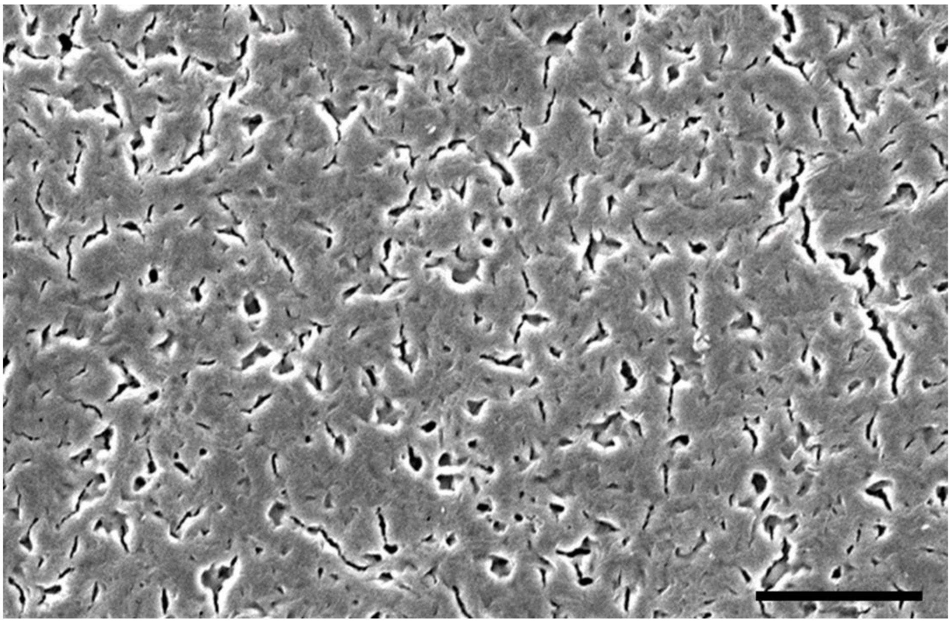

Supplementary Figure 39 | FE-SEM image of MMMs containing NUS-24 nanosheets (Scale bar, 50 μm).

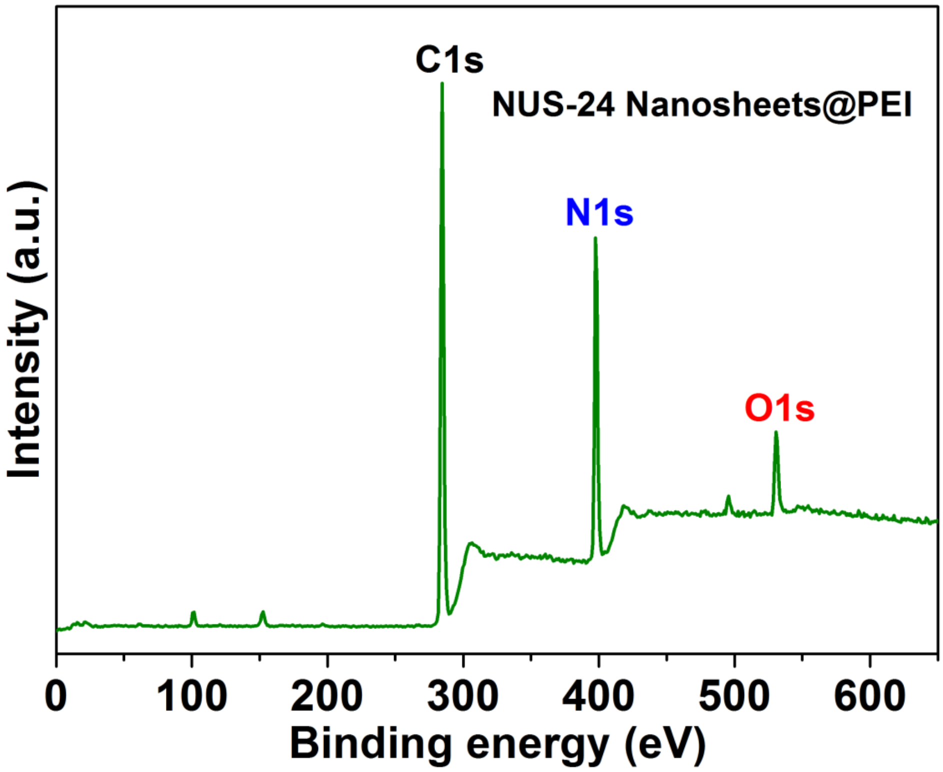

Supplementary Figure 40 | XPS spectra of MMMs containing NUS-24 nanosheets.

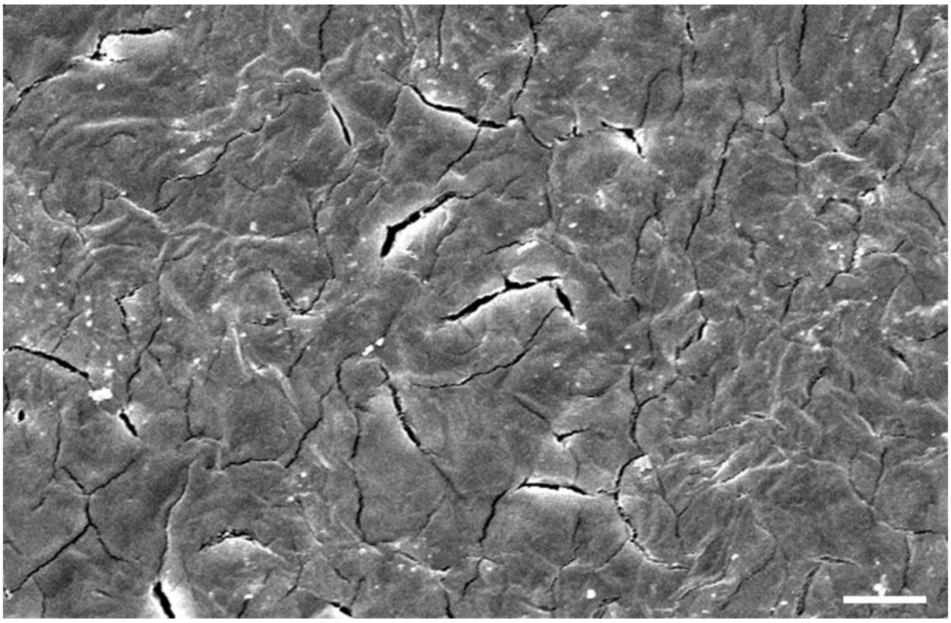

Supplementary Figure 41 | FE-SEM image of MMMs containing NUS-24 nanosheets after exposure to aqueous Fe^{3+} solutions (Scale bar, 10 μm).

Supplementary Table 5 Stability tests of NUS-24 bulk powder.

**Media**	**Solubility**	**Stability**
Air	-	Y
Water	N	Y
NaOH (8 M)	N	Y
HCl (6 M)	N	Y
H_2SO_4 (6 M)	N	Y
Tetrahydrofuran	N	Y
Acetone	N	Y
Dimethyl sulfoxide	N	Y
Dimethylformamide	N	Y
Dichloromethane	N	Y
Chloroform	N	Y
Ethanol	N	Y
Acetonitrile	N	Y
Toluene	N	Y
Dioxane	N	Y
Hexane	N	Y

N, not soluble; Y, stable. Stability was tested by microscopic observations and FTIR spectroscopy of the sample after being kept in the corresponding media for one month.

Supplementary Table 6 The quenching percentage and quenching K_{SV} constant of NUS-24 nanosheets titrated with different metal ion solutions.

**Metal ions**	**Quenching percentage (%)**	**K_{sv} Constant (M^{-1})**
Li^{+}	2.0	36
Na^{+}	2.8	67
K^{+}	3.8	101
Ag^{+}	4.5	113
Mg^{2+}	3.4	96
Ca^{2+}	1.7	36
Sr^{2+}	9.2	261
Ba^{2+}	2.3	56
Zn^{2+}	1.9	38
Cd^{2+}	4.7	106
Cu^{2+}	54.1 ± 4	3242 ± 500
Ni^{2+}	3.0	53
Co^{2+}	51.1 ± 4	4387 ± 600
Mn^{2+}	41.4 ± 3	1984 ± 400
Pb^{2+}	3.7	99
Al^{3+}	7.2	188
Ln^{3+}	4.5	95
Fe^{3+}	91.7 ± 4	27214 ± 1500
Cr^{3+}	13.0	406
Ce^{3+}	20.1	653
Eu^{3+}	1.1	63
Ga^{3+}	6.5	189
Tb^{3+}	7.5	234
Er^{3+}	6.9	183
VO^{2+}	28.9	1172
Ce^{4+}	9.3	245

Supplementary Table 7 The quenching percentages and quenching K_{SV} constants of TPE-1, TPE-2, TPE, pyrene and NUS-24 bulk powder titrated with different metal ion solutions.

	**Metal ions**	**Quenching percentage (%)**	**K_{sv} constant (M^{-1})**
TPE-1	Fe^{3+}	47.3 ± 3	2011 ± 200
	Cu^{2+}	13.6 ± 2	410 ± 50
	Mn^{2+}	39.9 ± 3	1460 ± 200
	Co^{2+}	5.7	153
TPE-2	Fe^{3+}	42.3 ± 3	1659 ± 150
	Cu^{2+}	32.1 ± 2	1201 ± 100
	Mn^{2+}	28.7 ± 3	955 ± 100
	Co^{2+}	0.6	7
TPE	Fe^{3+}	61.7 ± 4	3632 ± 400
	Cu^{2+}	33.6 ± 2	1293 ± 100
	Mn^{2+}	38.0 ± 3	1414 ± 100
	Co^{2+}	4.7	141
Pyrene	Fe^{3+}	48.9 ± 3	2296 ± 300
	Cu^{2+}	30.3 ± 2	1081 ± 100
	Mn^{2+}	14.3 ± 2	435 ± 50
	Co^{2+}	3.7	98
NUS-24 Bulk	Fe^{3+}	82.4 ± 4	10617 ± 500
	Cu^{2+}	40.4 ± 3	1744 ± 150
	Mn^{2+}	26.1 ± 2	886 ± 100
	Co^{2+}	30.4	1057

Supplementary Table 8 The values of $K_{sv}(Fe^{3+})/K_{sv}(Cu^{2+})$ and $K_{sv}(Fe^{3+})/K_{sv}(Mn^{2+})$ for TPE-1, TPE-2, TPE, pyrene, NUS-24 bulk and NUS-24 nanosheets.

	$K_{sv}(Fe^{3+})/K_{sv}(Cu^{2+})$	$K_{sv}(Fe^{3+})/K_{sv}(Mn^{2+})$
TPE-1	4.90	1.38
TPE-2	1.38	1.74
TPE	2.81	2.57
Pyrene	2.12	5.28
NUS-24 bulk powder	6.09	11.98
NUS-24 nanosheets	8.39	13.72

Supplementary References

1 Hu, R. *et al.* Luminogenic materials constructed from tetraphenylethene building blocks: Synthesis, aggregation-induced emission, two-photon absorption, light refraction, and explosive detection. *J. Mater. Chem.* **22**, 232-240 (2012).

2 Wang, J. *et al.* Ethynyl-capped hyperbranched conjugated polytriazole: Click polymerization, clickable modification, and aggregation-enhanced emission. *Macromolecules* **45**, 7692-7703 (2012).

3 Frisch, M. J. T., G. W.; Schlegel, H. B.; Scuseria, G. E.; Robb, M. A.; Cheeseman, J. R.; Zakrzewski, V. G.; Montgomery, J. A.; Stratmann, R. E.; Burant, J. C.; Dapprich, S.; Millam, J. M.; Daniels, A. D.; Kudin, K. N.; Strain, M. C.; Farkas, O.; Tomasi, J.; Barone, V.; Cossi, M.; Cammi, R.; Mennucci, B.; Pomelli, C.; Adamo, C.; Clifford, S.; Ochterski, J.; Petersson, G. A.; Ayala, P. Y.; Cui, Q.; Morokuma, K.; Malick, D. K.; Rabuck, A. D.; Raghavachari, K.; Foresman, J. B.; Cioslowski, J.; Ortiz, J. V.; Stefanov, B. B.; Liu, G.; Liashenko, A.; Piskorz, P.; Komaromi, I.; Gomperts, R.; Martin, R. L.; Fox, D. J.; Keith, T.; Al-Laham, M. A.; Peng, C. Y.; Nanayakkara, A.; Gonzalez, C.; Challacombe, M.; Gill, P. M. W.; Johnson, B. G.; Chen, W.; Wong, M. W.; Andres, J. L.; Head-Gordon, M.; Replogle, E. S.; Pople, J. A. *Gaussian 09, Gaussian Inc.*, (2009).

4 Rappé, A. K. *et al.* Uff, a full periodic table force field for molecular mechanics and molecular dynamics simulations. *J. Am. Chem. Soc.* **114**,

10024-10035 (1992).

5 Rappe, A. K. & Goddard III, W. A. Charge equilibration for molecular dynamics simulations. *J. Phys. Chem.* **95**, 3358-3363 (1991).

6 Elstner, M. *et al.* Self-consistent-charge density-functional tight-binding method for simulations of complex materials properties. *Phys. Rev. B* **58**, 7260 (1998).

7 Becke, A. D. Density functional thermochemistry. Iii. The role of exact exchange. *J. Chem. Phys.* **98**, 5648-5652 (1993).
